# Dominant control of agriculture and irrigation on urban heat island in India

**DOI:** 10.1038/s41598-017-14213-2

**Published:** 2017-10-25

**Authors:** Rahul Kumar, Vimal Mishra, Jonathan Buzan, Rohini Kumar, Drew Shindell, Matthew Huber

**Affiliations:** 1 Civil Engineering, Indian Institute of Technology Gandhinagar, Gujarat, 382355 India; 2 Earth, Atmospheric and Planetary Sciences, Purdue University, West Lafayette, IN 47907 USA; 3 UFZ-Helmholtz Centre for Environmental Research, Leipzig, Germany; 4 School of Environment, Duke University, Durham, USA

## Abstract

As is true in many regions, India experiences surface Urban Heat Island (UHI) effect that is well understood, but the causes of the more recently discovered Urban Cool Island (UCI) effect remain poorly constrained. This raises questions about our fundamental understanding of the drivers of rural-urban environmental gradients and hinders development of effective strategies for mitigation and adaptation to projected heat stress increases in rapidly urbanizing India. Here we show that more than 60% of Indian urban areas are observed to experience a day-time UCI. We use satellite observations and the Community Land Model (CLM) to identify the impact of irrigation and prove for the first time that UCI is caused by lack of vegetation and moisture in non-urban areas relative to cities. In contrast, urban areas in extensively irrigated landscapes generally experience the expected positive UHI effect. At night, UHI warming intensifies, occurring across a majority (90%) of India’s urban areas. The magnitude of rural-urban temperature contrasts is largely controlled by agriculture and moisture availability from irrigation, but further analysis of model results indicate an important role for atmospheric aerosols. Thus both land-use decisions and aerosols are important factors governing, modulating, and even reversing the expected urban-rural temperature gradients.

## Introduction

Urbanisation is a complex phenomenon and is associated with some extreme environmental issues^1–4^. The world has witnessed rapid urbanization and by 2050, about 66% of the global population is expected to live in urban areas. Urban areas are hot spots of environmental changes, which alter biodiversity, water cycle, and climate^1^. In India, urban dwellers currently constitute well beyond 32% of total population and this is projected to increase substantially by 2050^5^. Consequently, it is important to understand the differing patterns of environmental change in rural and urban areas across India as well as their causes. The urban heat island (UHI: surface urban heat island is referred as UHI here onwards**)** effect is a phenomenon, in which surface temperature in urban areas is higher than surrounding non-urban areas^6,7^. The UHI effect is among the more significant changes to Earth’s surface climate induced by humans^8–12^. UHI can be affected by the radiation budget and land-use configuration of the local and urban-rural environment^13,14^. During heatwaves, UHI can enhance biophysical hazards such as heat stress, air pollution and other public health related issues^15^ including heat related mortality^16–19^, which are projected to become even more important in the future^20^.

India has witnessed rapid urbanization during the last few decades that is likely to continue in the future^20^. Recently, the government of India announced the development of 100 smart cities which will witness rapid growth in urban infrastructure and population, leading to increases in the UHI intensity with implications for temperature extremes, public health, and energy demands for residential cooling. Moreover, the frequency of heatwaves has increased in India^21,22^ and intensification of future severe heatwaves in India could lead to higher heat stress and mortality^23^. Despite a rapid urban growth in India, the major driving factors of surface UHI intensity have largely been unexplored in previous studies^14,24,25^.

Using simulations from the CLM and satellite observations, for the first time, we demonstrate conclusively that the agriculture and irrigation in India are the two main drivers of UHI, which have policy implications as both urbanization and agriculture intensification is likely to increase in the future. We investigate the dominant controls on UHI across 89 cities that are planned to be developed as smart cities in India. First, we estimate UHI based on 8-day LST composite data from Terra and Aqua sensors of the Moderate Resolution Spectroradiometer (MODIS) for the period of 2000–2014 for Terra and 2003–2014 for Aqua platforms. UHI intensity was estimated for pre-monsoon (February to May) and post-monsoon (October to January) periods. We excluded the monsoon season (June to September) from the analysis due to extensive cloudiness and unavailability of quality data of LST. UHI intensity was calculated as a difference between LST of urban-core and surrounding non-urban areas. The distance to the surrounding non-urban areas from the urban-core varied with the size of urban areas (Supplemental Table S1). UHI intensity was calculated for each 8-day LST composite in the pre and post monsoon seasons.

## Results and Discussion

### Day and night-time difference in surface temperature between urban and non-urban areas

**Figure 1:**
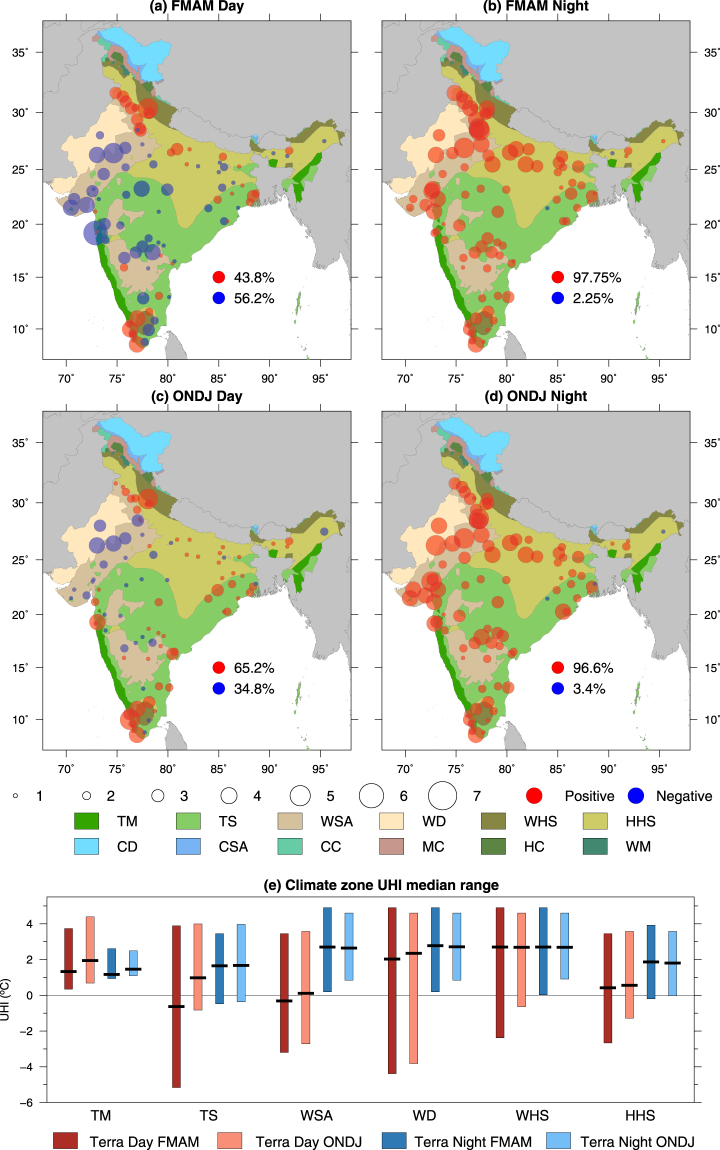
Day and night time Urban Heat Island (UHI) in the pre and post monsoon seasons in India for the 89 largest out of 100 urban areas that are planned as Smart Cities. Median difference (Urban Heat Island, UHI) between mean land surface temperature (LST, °C) in *urban-core* and mean LST for *non-urban* areas observed from the MODIS Terra satellite for the pre-monsoon (February to May, FMAM) and post-monsoon (October to January, ONDJ) seasons (**a**–**d**), and (**e**) range and median UHI (°C) for each climate zone in the pre and post monsoon seasons from Terra sensor. The size of circles shows magnitude of UHI. Red circles show UHI is positive while blue circles show that UHI is negative. Background colour in (**a**–**d**) represents geographic extent of the selected climate zones in India. The climate zones in consideration are Tropical Monsoon (TM), Tropical Savannah (TS), Warm Semi-arid (WSA), Warm Desert (WD), Warm Humid Subtropical (WHS), Hot Humid Subtropical (HHS), Cold Desert (CD), Cold Semi-arid (CSA), Cool Continental (CC), Mediterranean Continental (MC), Humid Continental (HC) and Warm Mediterranean (WM). Figure was created using Generic Mapping Tools version 5.4.2 (GMT: http://gmt.soest.hawaii.edu).

Median UHI intensity in the pre-monsoon (FMAM) season during daytime shows a large spatial and temporal variability (Fig. 1). UHI intensity is negative (i.e. Urban Cool Island, UCI) during daytime for more than half of the urban areas in India in the pre-monsoon season (Fig. 1a). Urban areas with UCI phenomenon were largely located in the western and central parts of India. However, daytime UHI intensity was positive during the pre-monsoon season (FMAM) for urban areas in the Gangetic Plain, north-western India (Punjab and Haryana), and southern tip of the west coast. We find that during the pre-monsoon season, a majority of urban areas shows UCI phenomenon with intensity varied between 1 and 5 °C (Fig. 1a,b). In contrast, during the post-monsoon season (ONDJ), more than 65% of urban areas show the UHI effect. Nevertheless, urban areas located in the semi-arid and arid regions of western India continued to show UCI phenomenon (Fig. 1c). During the daytime, we find consistent patterns of UHI intensity for 89 cities across India from both Terra and Aqua sensors of MODIS (Fig. S3). Seasonal variability and causes of the daytime UCI phenomenon remain largely unexplored in India^14^.

More than 95% of urban areas showed UHI phenomenon in night-time during the pre and post monsoon seasons (Fig. 1b,d). The UHI intensity varied between 1 and 5 °C while the median UHI intensity ranged between 1.5 and 3 °C for urban areas located in the different climatic zones in India (Fig. 1e). Both the magnitude and spatial variability of UHI intensity were consistent among Terra and Aqua sensors indicating the robustness of urban areas being warmer during night than their surroundings (Fig. 1, Fig. S3). We find that urban areas located in the semi-arid western India show higher UHI intensity in night during the both pre and post monsoon seasons. Impervious materials in the urban-core region has high thermal capacity in comparison to surrounding non-urban areas, which results in larger long-wave radiation from urban areas. Therefore, UHI during night-time is strongly correlated to the degree of imperviousness in urban areas as observed previously, for example, in Delhi^26^, New York City^27^ and the Twin Cities^28^. UHI intensity is more prominent during night-time due to a relatively higher warming of minimum temperature than maximum temperature in urban areas, which results in a lower diurnal range of temperature than surrounding non-urban areas^29^. Notably, this robust UHI intensity of 1 to 5 °C may have serious implications for public health, especially during heatwaves in urban India. For example, warmer night-time temperatures can worsen the severity of heatwaves^30^, which can cause a higher risk of heatwave related mortality^21^.

### Monthly variation in Urban Heat/Cool Island

**Figure 2:**
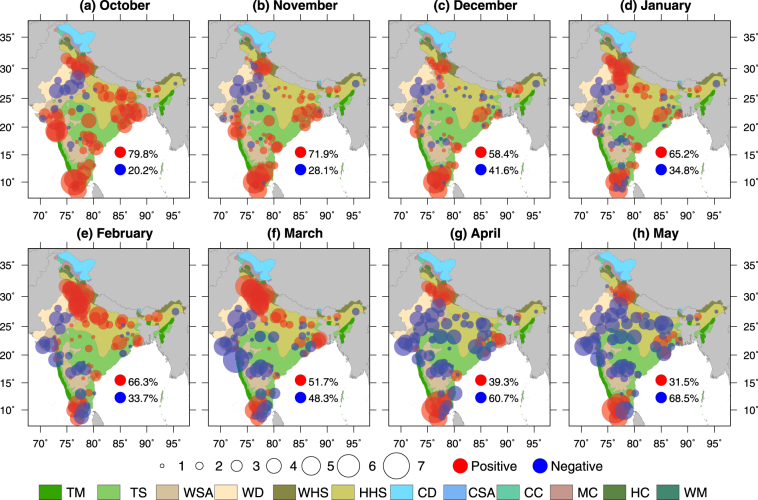
Monthly variation in day-time UHI in the pre and post monsoon seasons. (**a**–**d**) Day-time UHI (°C) for the post monsoon season (October–January), (**e**–**h**) day-time UHI for the pre-monsoon season (February–May). All the values were estimated for LST and NDVI data from MODIS Aqua sensor. The red and blue colours indicate positive and negative values of UHI. The size of the circles represents the intensities in °C. Figure was created using Generic Mapping Tools version 5.4.2 (GMT: http://gmt.soest.hawaii.edu).

To investigate the causes of UHI and UCI during the night and day times, respectively, we analysed monthly variability in land surface temperature in urban and surrounding non-urban areas. The number of urban areas showing UHI phenomenon is generally larger than that of UCI phenomenon during the post monsoon season (Fig. 2). For instance, just after the monsoon season in the month of October, about 80% of urban areas show strong day-time UHI intensity that varied between 1 and 6 °C (Fig. 2a). The other 20% of the total urban areas showed UCI phenomenon in October, which are largely centred in the semi-arid and arid region of the western India (Fig. 2a).

Daytime variability of the UHI/UCI phenomenon during the pre-monsoon season shows contrasting results than those were obtained for the post monsoon season (Fig. 2e–h). During the initial phase of the pre-monsoon season, the number of urban areas showing daytime UHI phenomenon are generally higher than those of the UCI phenomenon (Fig. 2e,f). For instance, 66 and 51% of the total 89 urban areas show the UHI effect in February and March, and most of them are located in the Indo-Gangetic Plain, north-west India, and the south-west coastal regions of India. Similar to the results obtained for the post-monsoon season, we find that urban areas with the UCI phenomenon are largely located in the semi-arid western India during the pre-monsoon season. Moreover our analysis shows that the number of urban areas with UCI phenomenon, and their UCI intensity substantially increases from March onwards (Fig. 2f). More than 60% of the total urban areas show the UCI phenomenon with intensity of 1-5 °C in the month of April (Fig. 2g). The number of urban areas showing the UCI phenomenon further increases to about 70% of the total urban areas in the month of May (Fig. 2h), when air and surface temperature are at their peaks due to the limited availability of moisture and crops in the surrounding non-urban areas. In contrast, during night-time, more than 90% of urban areas show the UHI phenomenon for all the months during the pre and post monsoon seasons (Figs S4 and S5), which is largely due to high thermal capacity of urban areas.

### Influence of Vegetation and Irrigation on UHI/UCI intensity

**Figure 3:**
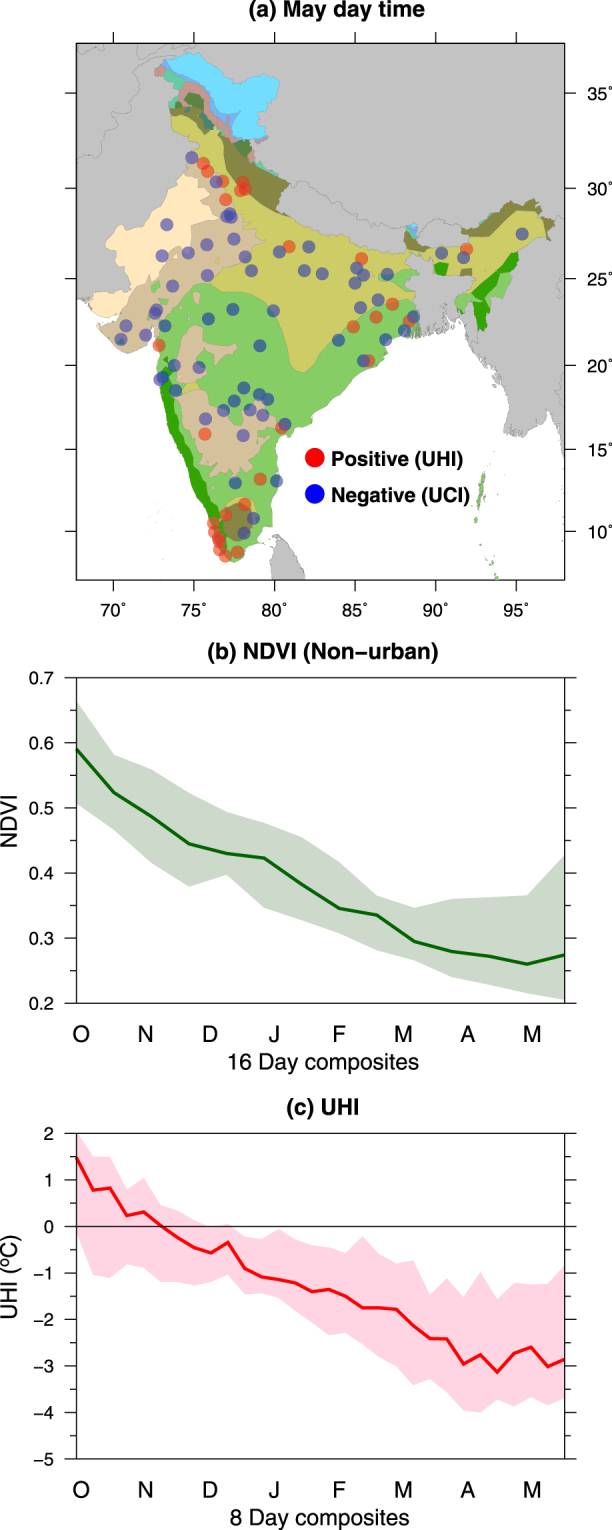
Influence of vegetation on UHI/UCI intensity in urban areas in India. (**a**) Location of urban areas that show Urban Cool Island (UCI) during the month of May (**b**) Monthly variation in NDVI of surrounding non-urban areas for urban areas that show negative day-time UHI or urban cool island in May (in Fig. 2h), and (**c**) monthly variation in day time UHI in May for urban areas shown in blue in (**a**). Figure was created using Generic Mapping Tools version 5.4.2 (GMT: http://gmt.soest.hawaii.edu).

To evaluate and quantify the linkage between vegetation in non-urban areas and the UHI/UCI phenomena we use 16-day Normalized Difference Vegetation Index (NDVI) from the MODIS Terra sensor (Fig. 3). As the majority of urban areas show the UCI effect during May, we selected these urban areas and identify the linkage between UCI and vegetation, mostly in the form of agriculture (as expressed by NDVI) in the surrounding non-urban areas (Fig. 3a–c). Most of the non-urban areas are dominated by agriculture, with more than 70 (out of total 89) non-urban areas have higher than 50% of total land cover as agriculture areas (Supplemental Table 3). The Rabi season (November to March) is a key cropping season in India that mostly relies on irrigation either from groundwater or surface water. We find that all the urban areas (about 70% of the total) that showed the UCI phenomenon in May (Figs 2 and 3a), NDVI in the surrounding non-urban areas declines substantially from October to May period (Fig. 3b). This decline in NDVI in the non-urban area is related to harvesting of crops by the end of March during the Rabi season. Thus, our analysis show the dominant role of vegetation (crop) cover in the surrounding non-urban areas in modulating the UHI/UCI phenomenon across India during the peak summer month of May (Fig. 3c). We note that the night-time UHI intensity is not so strongly associated with the vegetation variability in non-urban areas. However, the decline in NDVI reduces UHI intensity during the pre-monsoon period (Fig. S4i,j).

Urban areas located in the highly irrigated region (Indo-Gangetic Plain) and north-west India (Haryana and Punjab) show UHI intensity of 3–5 °C (Fig. 1b,d). The UHI and UCI phenomena in the Indo-Gangetic Plain and semi-arid western India, respectively indicate the role of moisture and vegetation on the surface temperature variability between the urban-core and the surrounding non-urban areas. During the summer season (April and May), when air temperature is at the peak in India, land surface temperature becomes much hotter than that of the post-monsoon season in absence of the agricultural crops. Moreover, amount of moisture and vegetation in non-urban areas are also limited as crops are largely harvested by the end of March and soil moisture is depleted due to high atmospheric water demands.

**Figure 4:**
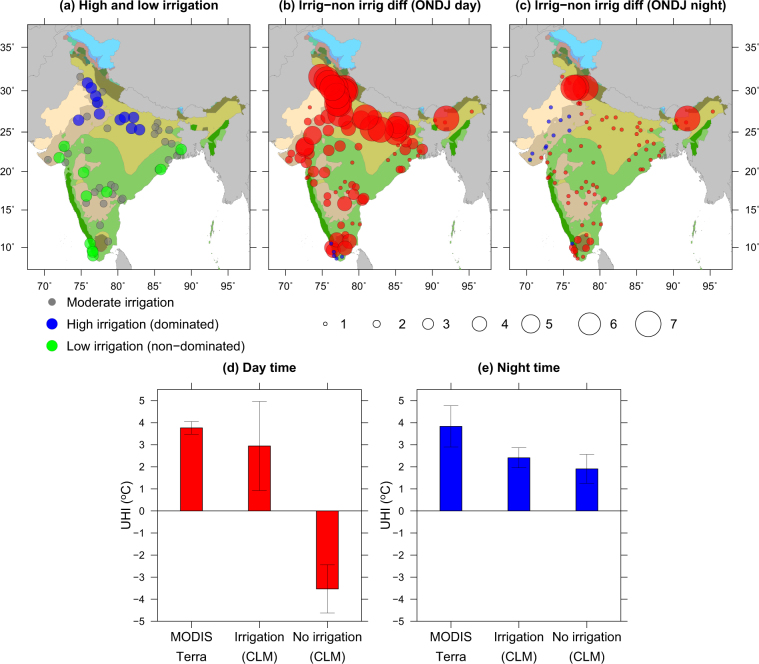
Influence of irrigation on UHI/UCI intensity in urban areas in India. (**a**) Location of urban areas that have high (blue)/low (green) fraction of irrigated area in their surrounding non-urban areas, (**b**) Difference in day-time UHI/UCI for with and without irrigation simulations from the CLM during the post-monsoon season, (**c**) same as (**b**) but for the night-time (**d**,**e**) comparison of day and night-time UHI from MODIS (Terra) and UHI estimated using the CLM runs for the with and without irrigation scenarios. Figure was created using Generic Mapping Tools version 5.4.2 (GMT: http://gmt.soest.hawaii.edu).

Using the high resolution (250 m) annual irrigated area maps^31^ and land cover data (56 m), we identify the urban areas that are predominantly located in the irrigated agriculture zone (Fig. 4a and Fig. S6). Non-urban areas which are dominated by irrigation (more than 80% of non-urban area is irrigated) show a contrasting response in their day-time UHI/UCI intensity relative to those where irrigation is less (less than 20% of non-urban area is irrigated). For instance, urban areas that are located in highly irrigated landscape show a considerable higher UHI, while areas that are located in less irrigated landscape show lower UHI intensity (Fig. 4d,e).

This observational based contrasting response of urban areas located in irrigated and non-irrigated landscapes is also corroborated with simulations from the Community Land Model (CLM) in which irrigation was turned on and off (Fig. 4b,c). Moreover, these model simulations that highlight the role of irrigation on UHI are consistent with observations from both Terra and Aqua sensors of MODIS (Fig. 4d,e). In both day and night-time, urban areas located in irrigation dominated landscapes show the UHI phenomenon (Fig. S7), while in the absence of agricultural crops and irrigation the urban areas show UCI phenomenon. We find that there is a moderate underestimation in UHI/UCI intensity in the CLM runs (Fig. 4d), which can be attributed to coarse resolution of the simulations. Overall our analysis highlights the role of vegetation and irrigation activities on the pattern of diurnal variations in UHI/UCI phenomena across Indian cities.

Other than agriculture and irrigation, climatic factors like precipitation and air temperature, as well as the size and population of urban areas can also influence the UHI intensity^11^. We find that Land surface temperature (LST) is directly related with the surface air temperature in a majority of urban areas across India (Figs S8–S10). Moreover, we also find that the size of urban areas are not directly associated with daytime UHI/UCI intensity, however, the night-time UHI intensity appears to have strong associate with the size of the urban-core region (Fig. S11).

### UHI variation and influence of atmospheric aerosol

We examined the plausibility of the alternative hypothesis that aerosol loading is substantially affecting the range of maximum and minimum surface temperatures using a Global Climate Model simulation. Mean monthly Aerosol Optical Depth (AOD) obtained from the Aqua and Terra platforms of MODIS show a high climatological AOD in the Indo-Gangetic Plain in the post monsoon season and in western India during the month of May (Figs S12–S13). We evaluate 8-year mean results from simulations with the GISS-E2 model^32^, which captures the spatial pattern of AOD fairly well, but is biased low relative to both ground-based and satellite observations over South Asia similar to most other composition-climate models^33^. We compare simulations driven by projected reference emissions with those in which emissions controls are put in place targeting sources rich in black carbon, similar to those in prior modeling^34^. Many aerosol species are affected by these emissions controls, but as the mixture is rich in black carbon and organic carbon is also treated as fairly absorbing (i.e. so-called ‘brown carbon’), the aerosol change is highly absorbing and likely reflects a fairly realistic mixture of the non-sulfate aerosols over India. We find that greater AOD in the reference case is highly collocated with a reduction in the daily maximum versus minimum temperature difference over most of South Asia (except snow and ice covered regions; Fig. S14). The global model is run at 2 × 2.5 degrees horizontal resolution and thus cannot resolve individual city centers. These results, however, suggest that a high loading of absorbing aerosols over Indian cities may indeed be at least partly responsible for the observed decreases in maximum minus minimum surface temperatures reported here. Day-time cooling in urban areas can also be attributed to the presence of atmospheric aerosols. Nevertheless, agriculture and irrigation are major factors that also contribute to day-time UCI in urban areas in India.

## Summary and Conclusions

India has witnessed unprecedented urban growth that is likely to increase in coming decades. Moreover, India experienced some of the most severe heat waves (e.g. 2015) that caused substantial mortality^21^. Understanding the role of urban and surrounding non-urban areas on the UHI intensity can provide policy insights that can help in urban planning and managing public health. We studied the surface UHI effect in 89 cities/urban areas that the Government of India considered for the development of smart cities. We, for the first time, evaluated the role of agriculture, irrigation, and atmospheric aerosols using the combination of *in-situ* and remotely sensed data and land surface and climate models. Our results from observations and climate models show that agriculture and irrigation are the two dominant drivers of UHI in India. Moreover, our modelling results demonstrate that a significant presence of atmospheric aerosols over the urban areas can influence UHI. The findings have implications for the future planning and decision making as a rapid growth in urbanization and irrigation is expected in the coming decades.

Rapidly growing urban areas in India show a robust UHI phenomenon in night with a UHI intensity of 1–5 °C in the pre and post monsoon seasons. Night-time UHI can have implications during the heat waves when day-time temperature is substantially high. Moreover, night time UHI and heat stress may increase further in response to climate warming^35^. Therefore, to avoid detrimental impacts of night-time UHI in rapidly urbanizing India, additional mitigation strategies are required, which can help in diminishing the impacts of climate warming^36^. Our results show that during the peak summer months (April to May), when heatwaves occur in India, daytime surface temperature of urban-core regions is lower than their surrounding non-urban areas. The UCI phenomenon is largely driven by agriculture and irrigation in non-urban areas, while atmospheric aerosols can also partially contribute to day-time urban cooling. Our study highlights the complexity of attributing the UHI/UCI phenomenon across India; and contributes to improved understanding on the role of human effects (irrigation) and agricultural activities as well as aerosol loading that have remained largely unexplored in the past.

## Methods

### Datasets

We used the Land Use Land Cover (LULC) map of year 2011 from National Remote Sensing Centre (NRSC), India along with the Moderate Resolution Imaging Spectroradiometer (MODIS) derived 500 m global urban extent data^37^ to map urban areas. Schneider *et al*.^37^ derived the urban extent map of global cities based on MODIS 500 m land cover data using a supervised classification scheme. We determined the high built-up portion or urban core (city centre or highly impervious area) of the city using the high resolution (56 m) data from NRSC. The urban and non-urban areas were demarcated from the MODIS urban extent map. We used 8-day Land Surface Temperature (LST) from both Terra and Aqua satellites of MODIS for the overlapping period of 2003 to 2014 to estimate surface urban heat island (UHI). Satellite derived LST captures a larger area and are relatively more reliable; however these satellite products also have (quality) issues mainly due to cloud contaminations.

We used day-time and night-time 8-day composite LST data from MODIS at 1 km spatial resolution which are derived from known emissivity in different bands of spectrum, as observed from the satellites in clear sky conditions. The MODIS products also provide the associated quality control flags that we used here to screen the quality-controlled LST data to further improve the reliability of the datasets. Observed surface air temperature at 2 m height was obtained from the Global Summary of Day (GSOD; ftp://ftp.ncdc.noaa.gov/pub/data/gsod/GSOD_DESC.txt accessed on 2 January 2017) dataset of the National Centers for Environmental Information (NCEI). The station data from the GSOD was checked for quality and inconsistencies, and only those stations that have continuous long-term data were selected for the analysis. More information on quality and consistency checks can be obtained from reference 4^4^.

To understand the variations in vegetation activities in urban and non-urban areas, 16-day Normalised Difference Vegetation Index (NDVI) available at 250 m spatial resolution was obtained from the MODIS Terra platform for the period 2003–2014. We compared difference in median NDVI values between urban core and non-urban with the UHI to understand the effect of vegetation cover. Apart from vegetation, aerosols and cloud fractions also affect the surface radiative budget and can subsequently impact urban heating processes. In many regions, urban areas experience a large concentration of atmospheric aerosol due to higher emissions^38,39^. We used the satellite derived monthly Aerosol Optical Depth (AOD) from Terra and Aqua platforms available at 10 km spatial resolution to understand seasonal variability of aerosols, which also influence UHI^40–43^. Furthermore, we performed numerical experiments using the NASA Goddard Institute for Space Studies atmospheric model^32^ to understand the spatial variation of aerosol loading (or AOD) and their effects on the UHI/UCI intensities across India.

### Estimation of Surface Urban Heat Island (UHI)

To compare the difference in LST between the urban core and non-urban areas (ΔT = T_u_ − T_r_), the non-urban area should lie sufficiently far away from the urban core to minimize the effects (spill-over). We considered a buffer of non-urban area for each urban area and the size of the buffer varied with the urban area. Urban areas were assigned with a 5 km width strip of non-urban area at distances that varied depending on the size of urban areas (Table S1). Moreover, we carefully checked for the topographical variations in the non-urban areas that could influence the estimated UHI effect. We analysed the LST data for the three regions: (1) high built-up area of the city or urban core, (2) low built up area inside the city boundary (urban area), and (3) a non-urban area that was at a distance away from the urban-core boundaries or rural belt (Table S1). These regions are marked as Zone 0, Zone 1, and Zone 2, respectively. In all the three zones, we excluded pixels covered with water bodies from the analysis while estimating mean LST over the urban or non-urban areas.

We considered the pre (February to May) and post (October-January) monsoon seasons for the analysis, and not included the monsoon (June to September) season as the LST data from the MODIS are often missing due to a high presence of cloud covers. Moreover, to reduce the effects of cloud cover in the pre and post monsoon seasons, we used 8-day composite of LST data for the common period of 2003 to 2014 in which both the Terra and Aqua sensors provided continuously the LST data. We used day and night LST products from both Terra and Aqua platforms to analyse the diurnal variations in LST. The satellite overpass time (in local time) is approximately around 10:30 and 22:30 hrs for the Terra satellite, and 01:30 and 13:30 hrs for the Aqua satellite. Therefore, these four observations from the Terra and Aqua satellites are used to capture the diurnal variation in LST.

We carefully evaluated the quality of MODIS based LST data sets. To reduce cloud contamination effects, LST images with more than 10% pixels of cloud cover, in any of the zones (e.g. Zone 0–2), were excluded from the computation of UHI. We used the associated quality flag information to compute the areal average as weighted mean LST for each zone (urban-core, urban area, and non-urban area) to derive the UHI intensity. The UHI intensity for every city was then estimated as the difference of weighted average LST between urban core (Zone 0) and the non-urban area (or Zone 2). The weighted mean LST of a zone $(\overline{LST})$ is determined as

1 $$\overline{LST}=\frac{{\sum }_{1}^{n}{w}_{p}LS{T}_{p}}{{\sum }_{1}^{n}{w}_{p}}$$

where, *w*_*p*_ and *LST*_*p*_ are weight and LST of the p^th^ pixel. The pixel-wise weight (*w*_*p*_) is based on the quality flag information taken as

2 $${w}_{p}=\{\begin{array}{l}3,\,good\,quality\,LST\\ 2,fair\,quality\,LST\\ 1,poor\,quality\,LST\end{array}$$

We also study the spatial variability in UHI/UCI across India based on their differences across different climate zones (Figure S1) that were derived based on the Köppen-Geiger climate classification map^44^. These climate regions are classified based on long-term monthly precipitation and temperature in the region. The dominant climate regions across India is classified as: Cold Desert (CD), Warm Mediterranean (WM), Cool Continental (CC), Tropical Monsoon (TM), Tropical Savannah (TS), Warm Semi-arid (WSA), Cold Semi-arid (CSA), Warm Desert Climate (WDC), Warm Humid Subtropical (WHS), Humid Continental (HC) and Hot Humid Subtropical (HHS).

### Community Land Model (CLM) Simulations

We use the Community Land Model Crop version 4 (CLM4)^45^ component model from the Community Earth System Model (CESM) developed by the National Center for Atmospheric Research (NCAR) to simulate land surface temperature. The surface UHI/UCI effects between irrigated and non-irrigated regions were estimated using surface temperature from CLM. Irrigation and the urban biome are incorporated into every grid cell in the India region of CLM4. We use a generic crop model for two different crop types, unmanaged rain fed and unmanaged irrigated crop. The control simulation only has unmanaged rain fed crops.We use this version rather than the more recent CLM4.5 because the irrigation model is operational for India in CLM4 only. We performed two simulations, one with cropland on and irrigation off and one with cropland on and irrigation on. Both sets of simulations were spun up using the CRUNCEP reanalysis driving dataset^46,47^, cycling over 1991–2010 for 500 years.

With these equilibrium initial conditions, we ran our experiment with CRUNCEP 2000-2014. We calculated drift in globally averaged surface temperature using −0.005 °C/century for our irrigation simulation, and 0 °C/century for the control run. Both simulations were executed with a 2° × 2° model resolution, and we output 4x daily values of ground surface rural, urban, and grid cell averaged temperatures to capture the diurnal cycle. A canyon model^6^ represents the urban environment in CLM4. Radiation, sensible, and latent heat fluxes are transferred between the walls, surface, and atmosphere of the canyon^48^.

## Electronic supplementary material

Supplementary Information 
