# Supplementary material for: Dominant control of agriculture and irrigation on urban heat island in India
Source: Sci Rep. 2017 Oct 25;7:14054. doi: 10.1038/s41598-017-14213-2 (PMC5656645; doi:10.1038/s41598-017-14213-2)
Supplement: Supplementary file 1 — Supplementary Information [file 41598_2017_14213_MOESM1_ESM.doc]

**Supplemental Information**

**Dominant control of agriculture and irrigation on urban heat island in India**

Rahul Kumar^1^, Vimal Mishra^1^, Jonathan Buzan^2^, Rohini Kumar^3^, Drew Schindell^4^, Matthew Huber^2^

1. Civil Engineering, Indian Institute of Technology Gandhinagar, Gujarat, India, 382355
2. Earth, Atmospheric and Planetary Sciences, Purdue University
3. UFZ-Helmholtz Centre for Environmental Research, Leipzig, Germany
4. School of Environment, Duke University, USA

*Corresponding Author:* Vimal Mishra, [vmishra@iitgn.ac.in](mailto:vmishra@iitgn.ac.in), +91-9687944337

**Supplementary Table 1.** The ranges of rural belts were decided based on the size of the cities derived from MODIS global urban extent map^1^.

| Type | Area range ( sq. m) | Rural range from boundary |
| --- | --- | --- |
| Tier I | > 40,00,00,000 | 45 - 50 km |
| Tier II | 20,00,00,000 - 40,00,00,000 | 40 - 45 km |
| Tier III | 10,00,00,000 - 20,00,00,000 | 35 - 40 km |
| Tier IV | 5,00,00,000 - 10,00,00,000 | 30 - 35 km |
| Tier V | < 5,00,00,000 | 25 - 30 km |

**Supplementary Table 2.** List of cities and their locations considered in the study. * are those cities which are also available with GSOD datasets.

| S. No. | City | Latitude | Longitude | Climate zone | Type |
| --- | --- | --- | --- | --- | --- |
| 1 | Bhiwandi | 19.2967 | 73.0631 | tropical monsoon | IV |
| 2 | Cochin | 9.9700 | 76.2800 | tropical monsoon | II |
| 3 | Kollam | 8.8800 | 76.6000 | tropical monsoon | V |
| 4 | Kottayam | 9.5800 | 76.5200 | tropical monsoon | V |
| 5 | Thrissur | 10.5200 | 76.2100 | tropical monsoon | V |
| 6 | Tiruvalla | 9.3850 | 76.5750 | tropical monsoon | V |
| 7 | Balasore* | 21.5000 | 86.9000 | tropical savannah | V |
| 8 | Bengaluru* | 12.9667 | 77.5667 | tropical savannah | I |
| 9 | Bhopal* | 23.2500 | 77.4167 | tropical savannah | II |
| 10 | Bhubaneshwar* | 20.2700 | 85.8400 | tropical savannah | IV |
| 11 | Bidar | 17.9000 | 77.5000 | tropical savannah | V |
| 12 | Chennai* | 13.0827 | 80.1257 | tropical savannah | I |
| 13 | Chittoor | 13.2000 | 79.1167 | tropical savannah | V |
| 14 | Cuttack | 20.2700 | 85.5200 | tropical savannah | III |
| 15 | Durgapur | 23.5500 | 87.3200 | tropical savannah | III |
| 16 | Guntur | 16.3008 | 80.4428 | tropical savannah | IV |
| 17 | Habra | 22.8300 | 88.6300 | tropical savannah | V |
| 18 | Haldia | 22.0300 | 88.0600 | tropical savannah | IV |
| 19 | Indore* | 22.7000 | 75.9000 | tropical savannah | II |
| 20 | Jamshedpur* | 22.8000 | 86.3000 | tropical savannah | III |
| 21 | Karimnagar | 18.2800 | 79.0600 | tropical savannah | V |
| 22 | Kolkata* | 22.5667 | 88.3667 | tropical savannah | I |
| 23 | Mumbai* | 19.1656 | 72.8846 | tropical savannah | I |
| 24 | Nagpur* | 21.1500 | 79.0900 | tropical savannah | II |
| 25 | Nashik | 20.0000 | 73.7800 | tropical savannah | III |
| 26 | Nizamabad | 18.6720 | 78.0940 | tropical savannah | V |
| 27 | Pune* | 18.5203 | 73.8567 | tropical savannah | II |
| 28 | Rourkela | 22.2492 | 84.8828 | tropical savannah | III |
| 29 | Sambalpur | 21.4700 | 83.9700 | tropical savannah | V |
| 30 | Surat* | 21.1700 | 72.8300 | tropical savannah | II |
| 31 | Thiruvananthapuram | 8.4875 | 76.9525 | tropical savannah | V |
| 32 | Tirunelvelli | 8.7300 | 77.7000 | tropical savannah | IV |
| 33 | Vadodara | 22.3000 | 73.2000 | tropical savannah | II |
| 34 | Vijayawada* | 16.5083 | 80.6417 | tropical savannah | IV |
| 35 | Warangal | 18.0000 | 79.5800 | tropical savannah | IV |
| 36 | Ahmedabad | 23.0300 | 72.5800 | warm semi-arid | I |
| 37 | Amritsar | 31.6400 | 74.8600 | warm semi-arid | III |
| 38 | Aurangabad | 19.8800 | 75.3200 | warm semi-arid | II |
| 39 | Badami | 15.9200 | 75.6803 | warm semi-arid | V |
| 40 | Bharatpur | 27.2200 | 77.4800 | warm semi-arid | IV |
| 41 | Bhavnagar | 21.7700 | 72.0000 | warm semi-arid | IV |
| 42 | Bijapur | 16.8300 | 75.7100 | warm semi-arid | IV |
| 43 | Delhi* | 28.61 | 77.23 | warm semi-arid |  |
| 44 | Faridabad | 28.4211 | 77.3078 | warm semi-arid | II |
| 45 | Gandhinagar | 23.2200 | 72.6800 | warm semi-arid | III |
| 46 | Gulbarga | 17.3333 | 76.8333 | warm semi-arid | IV |
| 47 | Gurgaon | 28.4700 | 77.0300 | warm semi-arid | II |
| 48 | Hyderabad | 17.3700 | 78.4800 | warm semi-arid | I |
| 49 | Jaipur* | 26.9000 | 75.8000 | warm semi-arid | II |
| 50 | Jalandhar | 31.3260 | 75.5760 | warm semi-arid | III |
| 51 | Junagadh | 21.5200 | 70.4700 | warm semi-arid | IV |
| 52 | Kota* | 25.1800 | 75.8300 | warm semi-arid | III |
| 53 | Kurnool | 15.8300 | 78.0500 | warm semi-arid | IV |
| 54 | Ludhiana | 30.9100 | 75.8500 | warm semi-arid | III |
| 55 | Nalgonda | 17.0500 | 79.2700 | warm semi-arid | IV |
| 56 | Panipat | 29.3900 | 76.9700 | warm semi-arid | IV |
| 57 | Patiala | 30.3400 | 76.3800 | warm semi-arid | IV |
| 58 | Rajkot | 22.3000 | 70.7833 | warm semi-arid | III |
| 59 | Udaipur | 24.5800 | 73.6800 | warm semi-arid | III |
| 60 | Ajmer | 26.4500 | 74.6400 | warm desert | III |
| 61 | Bikaner* | 28.0167 | 73.3119 | warm desert | IV |
| 62 | Jodhpur* | 26.2800 | 73.0200 | warm desert | II |
| 63 | Coimbatore* | 11.0183 | 76.9725 | warm humid subtropical | III |
| 64 | Dehradoon* | 30.3180 | 78.0290 | warm humid subtropical | III |
| 65 | Haridwar | 29.9560 | 78.1700 | warm humid subtropical | III |
| 66 | Madurai | 9.9000 | 78.1000 | warm humid subtropical | V |
| 67 | Allahabad | 25.4500 | 81.8500 | hot humid subtropical | II |
| 68 | Ambala | 30.3800 | 76.7800 | hot humid subtropical | III |
| 69 | Bhagalpur | 25.2500 | 87.0000 | hot humid subtropical | IV |
| 70 | Bihar_sharif | 25.1970 | 85.5180 | hot humid subtropical | V |
| 71 | Dhanbad | 23.7900 | 86.4300 | hot humid subtropical | I |
| 72 | Faizabad | 26.7800 | 82.1300 | hot humid subtropical | IV |
| 73 | Gaya* | 24.7500 | 85.0000 | hot humid subtropical | V |
| 74 | Goalpara | 26.4333 | 90.3667 | hot humid subtropical | V |
| 75 | Guwahati* | 26.1833 | 91.7333 | hot humid subtropical | III |
| 76 | Gwalior* | 26.2215 | 78.1780 | hot humid subtropical | III |
| 77 | Jabalpur* | 23.1667 | 79.9333 | hot humid subtropical | IV |
| 78 | Jhansi | 25.4486 | 78.5696 | hot humid subtropical | IV |
| 79 | Kanpur | 26.5000 | 80.3000 | hot humid subtropical | II |
| 80 | Lucknow* | 26.8000 | 80.9000 | hot humid subtropical | II |
| 81 | Muzaffarpur | 26.1200 | 85.4000 | hot humid subtropical | IV |
| 82 | Patna* | 25.6000 | 85.1000 | hot humid subtropical | II |
| 83 | Ranchi* | 23.3500 | 85.3300 | hot humid subtropical | IV |
| 84 | Roorkee | 29.8749 | 77.8899 | hot humid subtropical | IV |
| 85 | Salem | 11.6643 | 78.1460 | hot humid subtropical | IV |
| 86 | Tangla | 26.6571 | 91.9125 | hot humid subtropical | V |
| 87 | Tinsukia | 27.5000 | 95.3670 | hot humid subtropical | V |
| 88 | Tiruchirapalli* | 10.8050 | 78.6856 | hot humid subtropical | III |
| 89 | Varanasi* | 25.2800 | 82.9600 | hot humid subtropical | IV |

**Supplementary Table 3.** Percentage fraction of land use cover in the rural area (Zone 2) of each city in 2011. The Land Use Land Cover data is obtained from NRSC, India.

| S.No. | Latitude | Longitude | City | Built-up | Croplands | Forests | Grasslands | Scrublands | Swamps & Wastelands |
| --- | --- | --- | --- | --- | --- | --- | --- | --- | --- |
| 1 | 23.0 | 72.6 | Ahmedabad | 0.662 | 75.815 | 0.000 | 0.000 | 7.916 | 15.607 |
| 2 | 26.5 | 74.6 | Ajmer | 0.083 | 80.686 | 0.476 | 0.000 | 3.267 | 15.487 |
| 3 | 25.5 | 81.9 | Allahabad | 0.005 | 84.609 | 0.752 | 0.000 | 8.043 | 6.591 |
| 4 | 30.4 | 76.8 | Ambala | 2.694 | 54.515 | 29.038 | 5.449 | 6.744 | 1.560 |
| 5 | 31.6 | 74.9 | Amritsar | 4.607 | 93.916 | 0.020 | 0.000 | 0.576 | 0.881 |
| 6 | 19.9 | 75.3 | Aurangabad | 0.097 | 73.408 | 3.413 | 0.000 | 4.462 | 18.620 |
| 7 | 15.9 | 75.7 | Badami | 0.096 | 78.971 | 1.067 | 0.098 | 8.104 | 11.663 |
| 8 | 21.5 | 86.9 | Balasore | 0.000 | 80.167 | 14.259 | 0.002 | 3.943 | 1.630 |
| 9 | 13.0 | 77.6 | Bengaluru | 0.425 | 74.898 | 15.747 | 0.000 | 2.671 | 6.260 |
| 10 | 25.3 | 87.0 | Bhagalpur | 0.219 | 91.062 | 0.011 | 0.000 | 1.955 | 6.753 |
| 11 | 27.2 | 77.5 | Bharatpur | 0.493 | 96.050 | 0.056 | 0.000 | 0.721 | 2.679 |
| 12 | 21.8 | 72.0 | Bhavnagar | 0.044 | 72.539 | 6.155 | 0.000 | 9.014 | 12.248 |
| 13 | 19.3 | 73.1 | Bhiwandi | 0.036 | 22.373 | 39.999 | 0.000 | 26.942 | 10.650 |
| 14 | 23.3 | 77.4 | Bhopal | 0.010 | 68.465 | 25.628 | 0.000 | 2.245 | 3.652 |
| 15 | 20.3 | 85.8 | Bhubaneshwar | 0.000 | 89.272 | 2.294 | 0.000 | 1.041 | 7.393 |
| 16 | 17.9 | 77.5 | Bidar | 0.002 | 92.265 | 0.000 | 0.000 | 5.015 | 2.718 |
| 17 | 25.2 | 85.5 | Bihar_sharif | 1.172 | 92.661 | 0.000 | 0.000 | 1.088 | 5.078 |
| 18 | 16.8 | 75.7 | Bijapur | 0.043 | 78.327 | 0.000 | 0.000 | 0.562 | 21.068 |
| 19 | 28.0 | 73.3 | Bikaner | 0.000 | 43.074 | 0.031 | 0.000 | 28.008 | 28.886 |
| 20 | 13.1 | 80.1 | Chennai | 0.231 | 56.737 | 10.332 | 0.000 | 20.165 | 12.535 |
| 21 | 13.2 | 79.1 | Chittoor | 0.247 | 40.020 | 27.686 | 0.000 | 31.289 | 0.759 |
| 22 | 10.0 | 76.3 | Cochin | 0.607 | 40.076 | 52.360 | 2.314 | 0.000 | 4.642 |
| 23 | 11.0 | 77.0 | Coimbatore | 3.378 | 62.733 | 19.840 | 2.721 | 2.484 | 8.844 |
| 24 | 20.3 | 85.5 | Cuttack | 0.000 | 76.459 | 11.967 | 0.042 | 3.069 | 8.464 |
| 25 | 30.3 | 78.0 | Dehradoon | 0.013 | 14.784 | 51.113 | 18.798 | 8.810 | 6.482 |
| S.No. | Latitude | Longitude | City | Built-up | Croplands | Forests | Grasslands | Scrublands | Swamps & Wastelands |
| 26 | 28.6 | 77.2 | Delhi | 4.476 | 89.385 | 0.624 | 0.000 | 2.451 | 3.065 |
| 27 | 23.8 | 86.4 | Dhanbad | 0.196 | 72.941 | 9.368 | 0.010 | 6.141 | 11.345 |
| 28 | 23.6 | 87.3 | Durgapur | 0.505 | 79.212 | 12.361 | 0.136 | 1.190 | 6.596 |
| 29 | 26.8 | 82.1 | Faizabad | 0.000 | 92.173 | 0.461 | 0.000 | 3.977 | 3.389 |
| 30 | 28.4 | 77.3 | Faridabad | 3.139 | 90.515 | 0.348 | 0.000 | 2.602 | 3.396 |
| 31 | 23.2 | 72.7 | Gandhinagar | 0.959 | 87.894 | 1.088 | 0.000 | 8.691 | 1.368 |
| 32 | 24.8 | 85.0 | Gaya | 0.337 | 89.316 | 1.427 | 0.000 | 3.075 | 5.845 |
| 33 | 26.4 | 90.4 | Goalpara | 0.557 | 67.648 | 10.662 | 6.043 | 0.000 | 15.090 |
| 34 | 17.3 | 76.8 | Gulbarga | 0.109 | 95.370 | 0.000 | 0.000 | 2.415 | 2.106 |
| 35 | 16.3 | 80.4 | Guntur | 3.327 | 85.048 | 4.221 | 0.000 | 5.815 | 1.589 |
| 36 | 28.5 | 77.0 | Gurgaon | 4.177 | 84.272 | 4.281 | 0.000 | 3.257 | 4.013 |
| 37 | 26.2 | 91.7 | Guwahati | 0.113 | 61.519 | 25.127 | 4.485 | 0.000 | 8.755 |
| 38 | 26.2 | 78.2 | Gwalior | 0.064 | 62.390 | 11.602 | 0.000 | 10.692 | 15.252 |
| 39 | 22.8 | 88.6 | Habra | 0.000 | 96.971 | 0.000 | 0.512 | 0.041 | 2.476 |
| 40 | 22.0 | 88.1 | Haldia | 0.171 | 87.095 | 0.000 | 0.001 | 0.003 | 12.730 |
| 41 | 30.0 | 78.2 | Haridwar | 0.183 | 34.069 | 40.951 | 12.029 | 8.949 | 3.820 |
| 42 | 17.4 | 78.5 | Hyderabad | 0.350 | 74.870 | 3.933 | 0.000 | 15.306 | 5.541 |
| 43 | 22.7 | 75.9 | Indore | 0.035 | 80.640 | 14.332 | 0.000 | 3.412 | 1.582 |
| 44 | 23.2 | 79.9 | Jabalpur | 0.000 | 57.454 | 36.658 | 0.000 | 1.310 | 4.578 |
| 45 | 26.9 | 75.8 | Jaipur | 0.021 | 79.512 | 6.551 | 0.000 | 3.934 | 9.983 |
| 46 | 31.3 | 75.6 | Jalandhar | 3.108 | 61.491 | 25.047 | 0.283 | 7.032 | 3.039 |
| 47 | 22.8 | 86.3 | Jamshedpur | 0.270 | 66.230 | 21.156 | 0.024 | 2.520 | 9.799 |
| 48 | 25.4 | 78.6 | Jhansi | 0.567 | 71.815 | 10.469 | 0.000 | 8.235 | 8.913 |
| 49 | 26.3 | 73.0 | Jodhpur | 0.000 | 59.401 | 0.000 | 0.000 | 10.234 | 30.365 |
| 50 | 21.5 | 70.5 | Junagadh | 0.138 | 79.584 | 5.827 | 0.000 | 9.652 | 4.799 |
| 51 | 26.5 | 80.3 | Kanpur | 0.045 | 84.980 | 0.289 | 0.000 | 3.365 | 11.321 |
| 52 | 18.3 | 79.1 | Karimnagar | 2.404 | 75.447 | 5.402 | 0.000 | 13.021 | 3.725 |
| 53 | 22.6 | 88.4 | Kolkata | 1.608 | 90.305 | 0.000 | 0.132 | 0.137 | 7.818 |
| S.No. | Latitude | Longitude | City | Built-up | Croplands | Forests | Grasslands | Scrublands | Swamps & Wastelands |
| 54 | 8.9 | 76.6 | Kollam | 1.710 | 97.747 | 0.143 | 0.000 | 0.000 | 0.400 |
| 55 | 25.2 | 75.8 | Kota | 0.031 | 57.314 | 11.401 | 0.000 | 2.353 | 28.901 |
| 56 | 9.6 | 76.5 | Kottayam | 0.000 | 99.469 | 0.507 | 0.009 | 0.000 | 0.015 |
| 57 | 15.8 | 78.1 | Kurnool | 0.867 | 77.120 | 1.605 | 0.000 | 7.218 | 13.190 |
| 58 | 26.8 | 80.9 | Lucknow | 0.024 | 89.051 | 0.000 | 0.000 | 4.972 | 5.952 |
| 59 | 30.9 | 75.9 | Ludhiana | 4.712 | 71.377 | 5.039 | 0.594 | 9.883 | 8.395 |
| 60 | 9.9 | 78.1 | Madurai | 0.094 | 66.490 | 23.161 | 0.000 | 3.345 | 6.910 |
| 61 | 19.2 | 72.9 | Mumbai | 0.849 | 31.906 | 34.159 | 0.000 | 22.157 | 10.929 |
| 62 | 26.1 | 85.4 | Muzaffarpur | 0.548 | 95.502 | 0.000 | 0.000 | 1.101 | 2.849 |
| 63 | 21.2 | 79.1 | Nagpur | 0.039 | 55.844 | 30.493 | 0.000 | 8.825 | 4.800 |
| 64 | 17.1 | 79.3 | Nalgonda | 0.247 | 78.905 | 0.690 | 0.000 | 10.970 | 9.187 |
| 65 | 20.0 | 73.8 | Nashik | 0.060 | 41.755 | 30.958 | 0.000 | 15.661 | 11.565 |
| 66 | 18.7 | 78.1 | Nizamabad | 0.998 | 63.738 | 11.715 | 0.000 | 9.508 | 14.041 |
| 67 | 29.4 | 77.0 | Panipat | 3.042 | 92.523 | 0.469 | 0.000 | 2.566 | 1.401 |
| 68 | 30.3 | 76.4 | Patiala | 5.591 | 91.968 | 0.228 | 0.000 | 0.538 | 1.675 |
| 69 | 25.6 | 85.1 | Patna | 0.007 | 91.769 | 0.000 | 0.246 | 2.984 | 4.993 |
| 70 | 18.5 | 73.9 | Pune | 0.112 | 44.481 | 24.246 | 0.000 | 18.708 | 12.453 |
| 71 | 22.3 | 70.8 | Rajkot | 0.425 | 68.584 | 6.452 | 0.000 | 13.319 | 11.220 |
| 72 | 23.4 | 85.3 | Ranchi | 0.000 | 59.819 | 26.549 | 0.000 | 11.339 | 2.293 |
| 73 | 29.9 | 77.9 | Roorkee | 0.401 | 88.890 | 3.180 | 0.000 | 5.263 | 2.267 |
| 74 | 22.2 | 84.9 | Rourkela | 0.000 | 43.188 | 51.540 | 0.000 | 4.176 | 1.095 |
| 75 | 11.7 | 78.1 | Salem | 0.041 | 65.076 | 22.183 | 0.000 | 8.870 | 3.830 |
| 76 | 21.5 | 84.0 | Sambalpur | 0.000 | 57.815 | 26.905 | 1.665 | 4.521 | 9.094 |
| 77 | 21.2 | 72.8 | Surat | 1.591 | 80.461 | 0.000 | 0.000 | 13.330 | 4.618 |
| 78 | 26.7 | 91.9 | Tangla | 0.000 | 57.906 | 38.594 | 0.000 | 0.202 | 3.298 |
| 79 | 8.5 | 77.0 | Thiruvananthapuram | 0.145 | 60.189 | 36.622 | 0.471 | 0.000 | 2.573 |
| 80 | 10.5 | 76.2 | Thrissur | 0.593 | 82.451 | 7.388 | 1.662 | 0.000 | 7.906 |
| 81 | 27.5 | 95.4 | Tinsukia | 0.000 | 56.245 | 26.499 | 5.619 | 0.000 | 11.637 |
| S.No. | Latitude | Longitude | City | Built-up | Croplands | Forests | Grasslands | Scrublands | Swamps & Wastelands |
| 82 | 10.8 | 78.7 | Tiruchirapalli | 0.000 | 72.805 | 12.992 | 0.000 | 6.241 | 7.962 |
| 83 | 8.7 | 77.7 | Tirunelvelli | 0.033 | 57.210 | 24.799 | 0.175 | 4.417 | 13.366 |
| 84 | 9.4 | 76.6 | Tiruvalla | 1.340 | 94.685 | 3.668 | 0.170 | 0.000 | 0.138 |
| 85 | 24.6 | 73.7 | Udaipur | 0.150 | 50.986 | 31.152 | 0.000 | 0.590 | 17.122 |
| 86 | 22.3 | 73.2 | Vadodara | 0.746 | 76.409 | 5.167 | 0.000 | 9.854 | 7.825 |
| 87 | 25.3 | 83.0 | Varanasi | 0.027 | 84.180 | 0.040 | 0.000 | 11.858 | 3.894 |
| 88 | 16.5 | 80.6 | Vijayawada | 1.952 | 85.956 | 4.283 | 0.000 | 5.457 | 2.352 |
| 89 | 18.0 | 79.6 | Warangal | 1.140 | 73.081 | 5.648 | 0.000 | 15.125 | 5.006 |


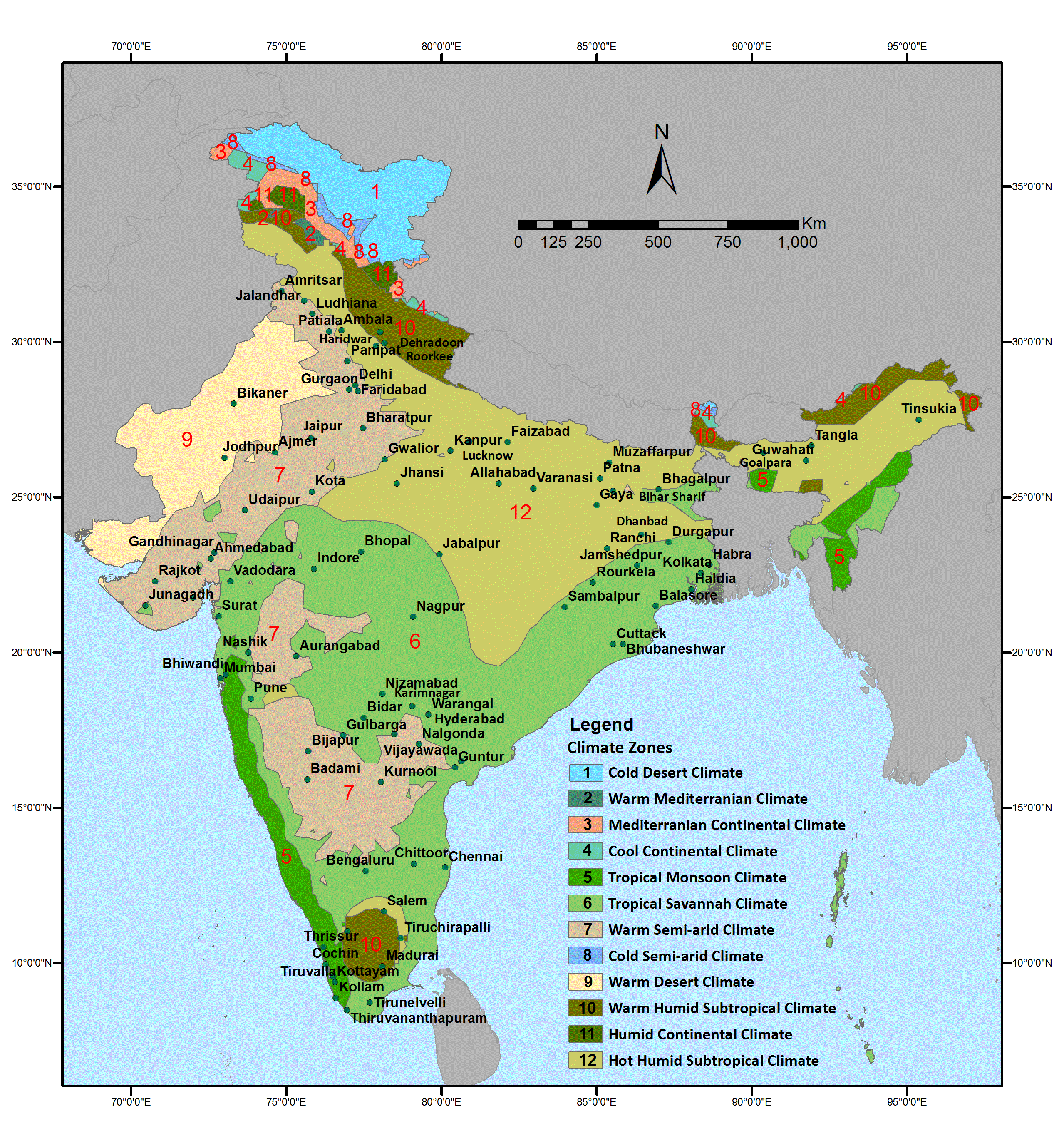


**Figure S1.** Climate zone map for India according to the modified Köppen-Geiger climate classification map^2^. The raster data for the map was obtained from Peel et al^2^, which is located at <http://www.hydrol-earth-syst-sci.net/11/1633/2007/hess-11-1633-2007-supplement.zip>. Figure was developed using ArcGIS version 10.5 https://www.arcgis.com/features/index.html.


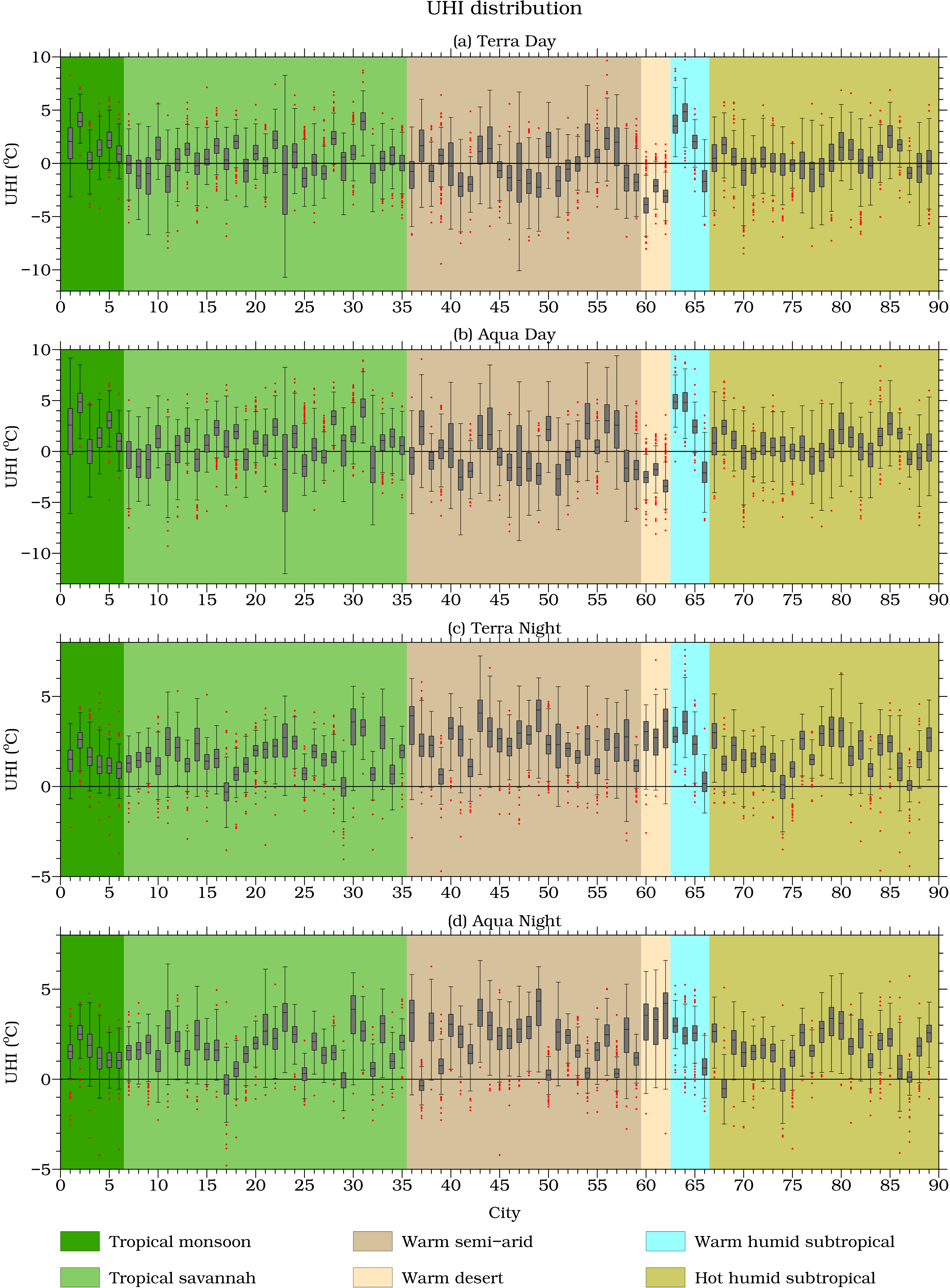


**Figure S2.** Distribution of UHI values for all cities in consideration. The sequence of cities are in the order of the list mentioned in supplementary data table 1. Figure was created using Generic Mapping Tools version 5.4.2 (GMT: http://gmt.soest.hawaii.edu).


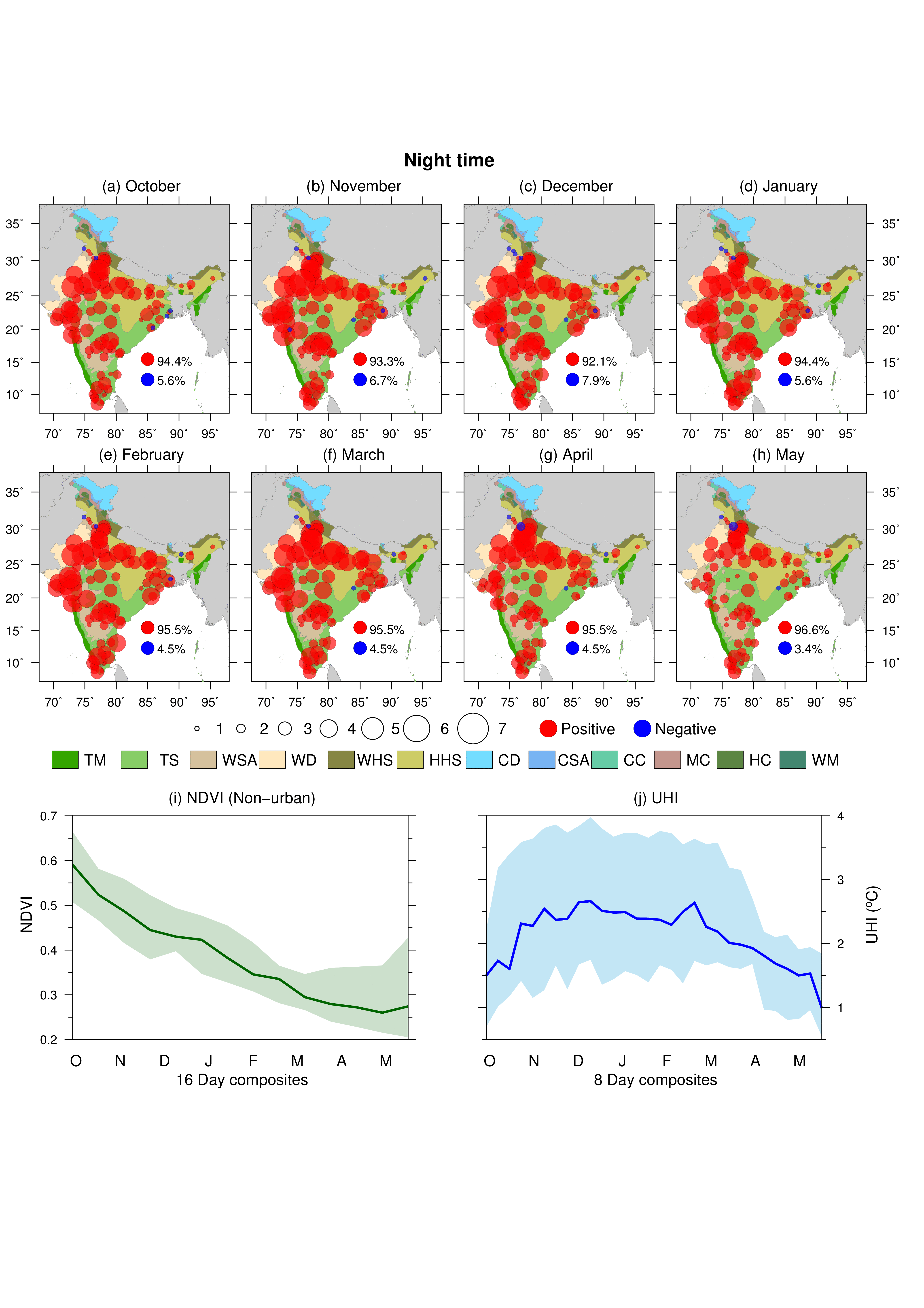


**Figure S3.** Same as Figure 3 but for nightime land surface temperature. Figure was created using Generic Mapping Tools version 5.4.2 (GMT: http://gmt.soest.hawaii.edu).


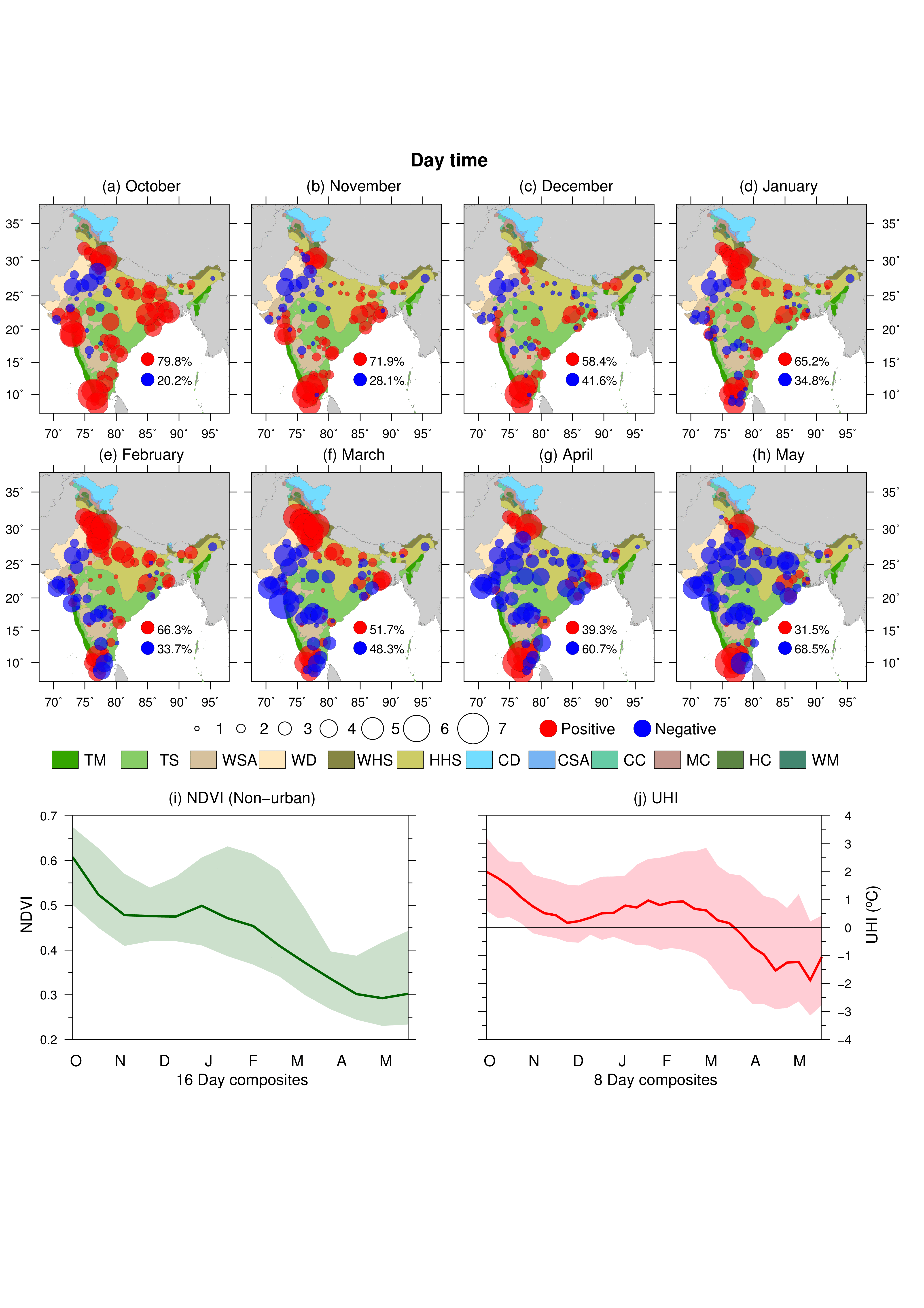


**Figure S4.** Same as Figure 3 but for all the urban areas instead of the urban areas that show UCI in the month of May. Figure was created using Generic Mapping Tools version 5.4.2 (GMT: http://gmt.soest.hawaii.edu).


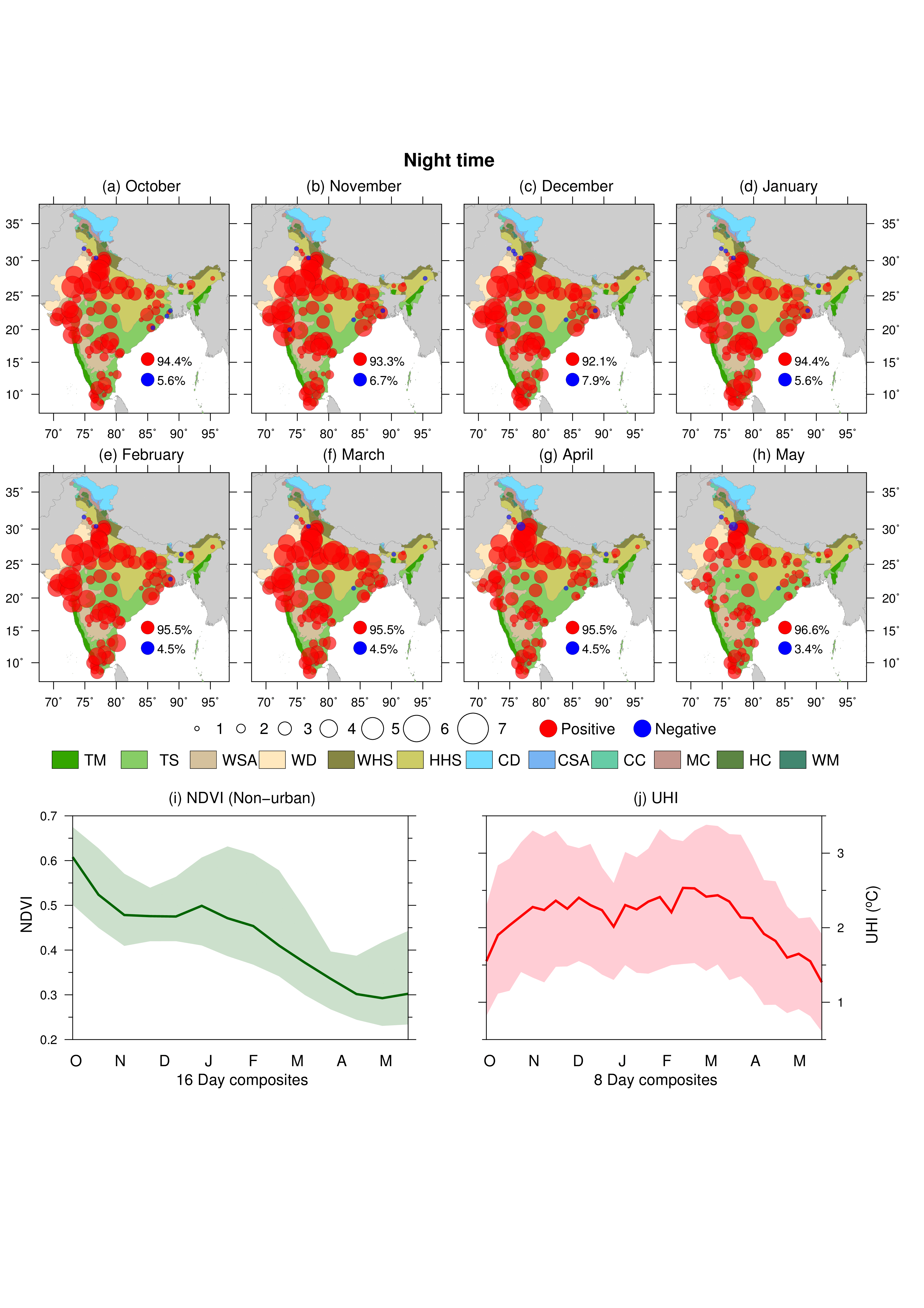


**Figure S5**. Same as Figure 3 but for nighttime and for all the urban areas instead of urban areas that show UCI during the month of May. Figure was created using Generic Mapping Tools version 5.4.2 (GMT: http://gmt.soest.hawaii.edu).


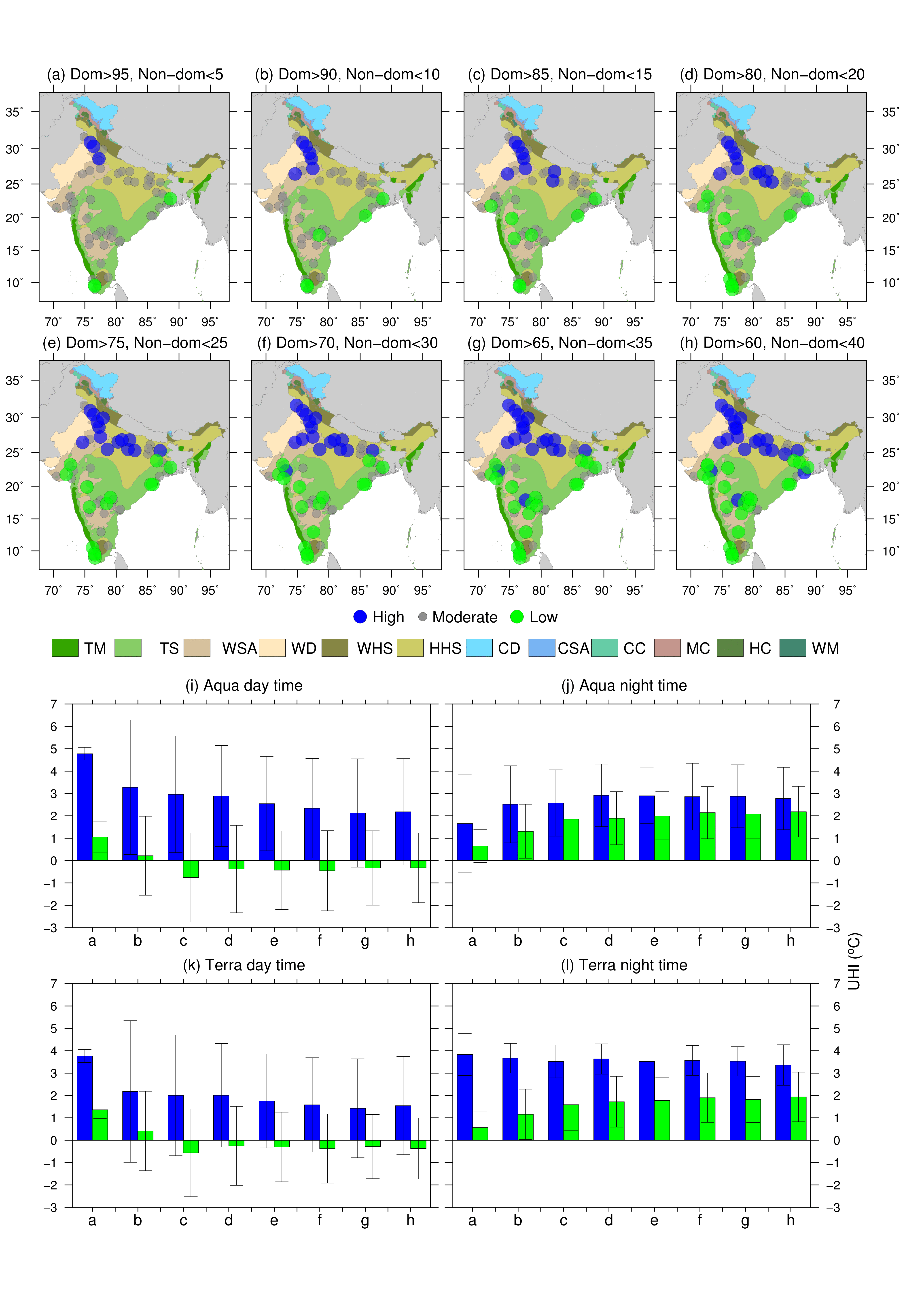


**Figure S6.** Same as Fig. 4 but for different threshold of irrigated area fraction. For instance in (a) urban areas are shown that have 95% and 5% of their surrounding non-urban areas are irrigated. (i-l) daytime and night time mean UHI/UCI intensity for selected urban areas in (a-h). Figure was created using Generic Mapping Tools version 5.4.2 (GMT: http://gmt.soest.hawaii.edu).


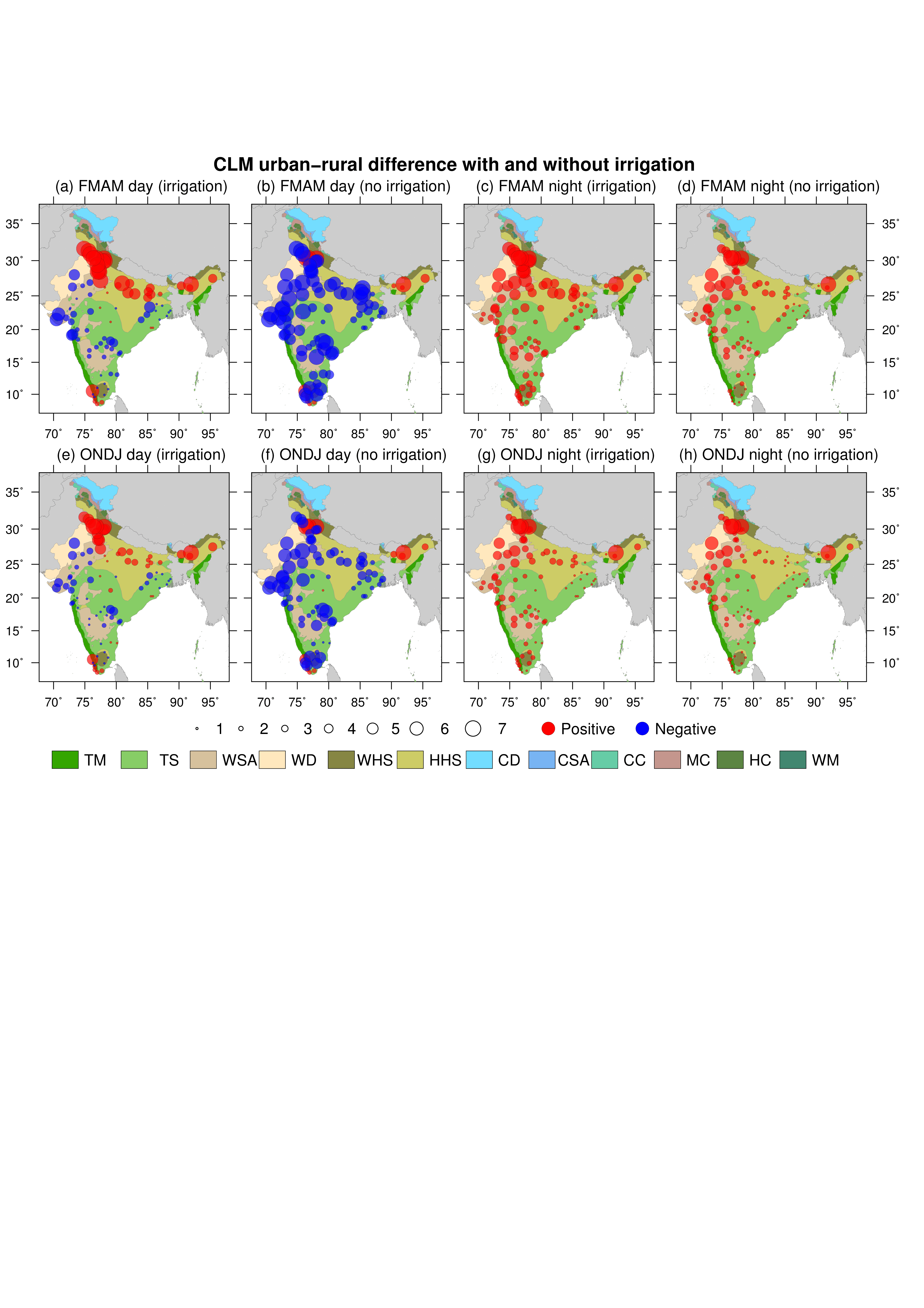


**Figure S7.**  Influence of irrigation on temperature difference between urban and rural regions based in CLM simulations. (a-b) Shows FMAM term day time temperature difference with and without irrigation;(c-d) Shows ONDJ term night time temperature difference with and without irrigation; (e-f) Shows ONDJ term day time temperature difference with and without irrigation; (g-h) Shows ONDJ term night time temperature difference with and without irrigation. Figure was created using Generic Mapping Tools (GMT). Figure was created using Generic Mapping Tools version 5.4.2 (GMT: http://gmt.soest.hawaii.edu).


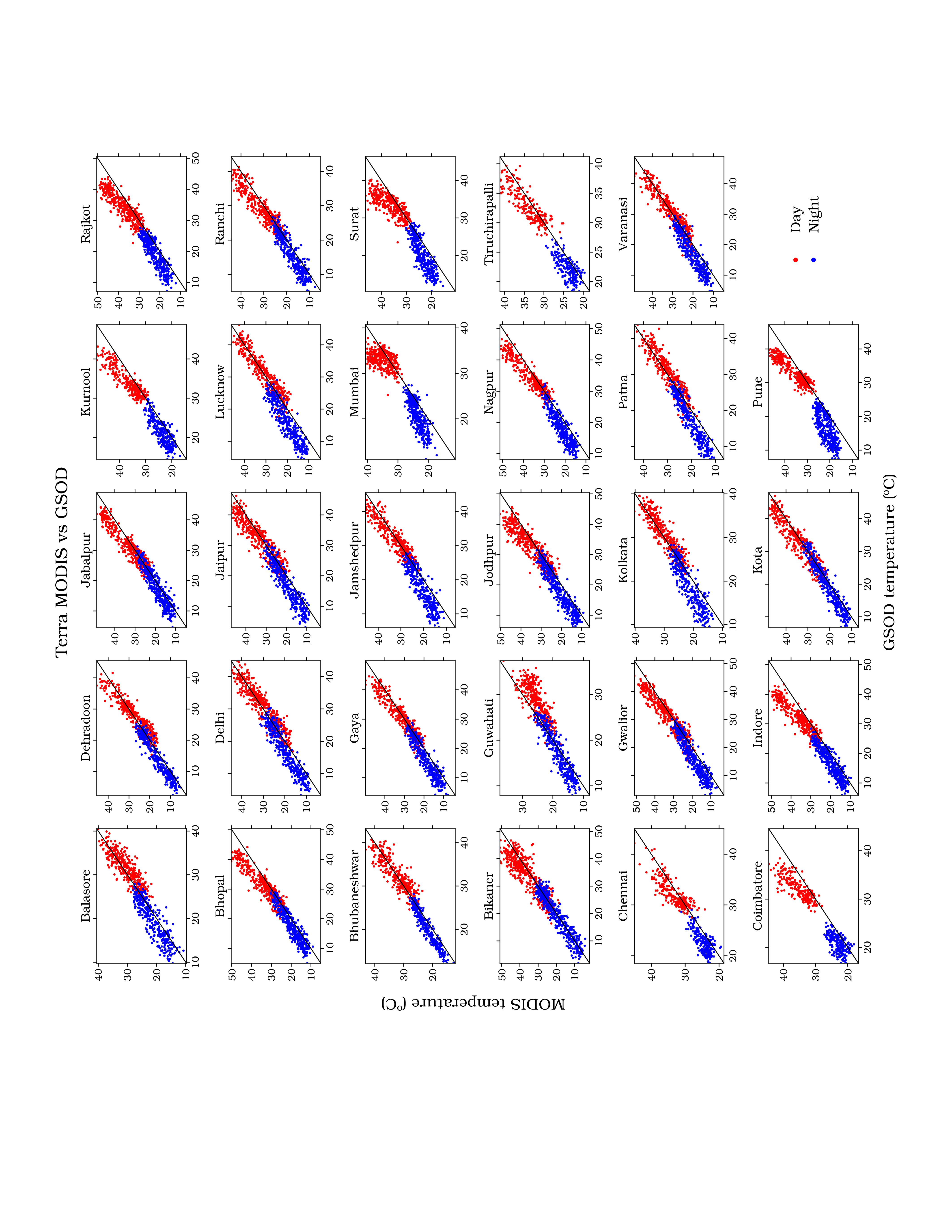


**Figure S8.** Relation between GSOD temperatures with respect to LST observed from Terra sensor for both day and night time represented by scatter plots. Figure was created using Generic Mapping Tools version 5.4.2 (GMT: http://gmt.soest.hawaii.edu).


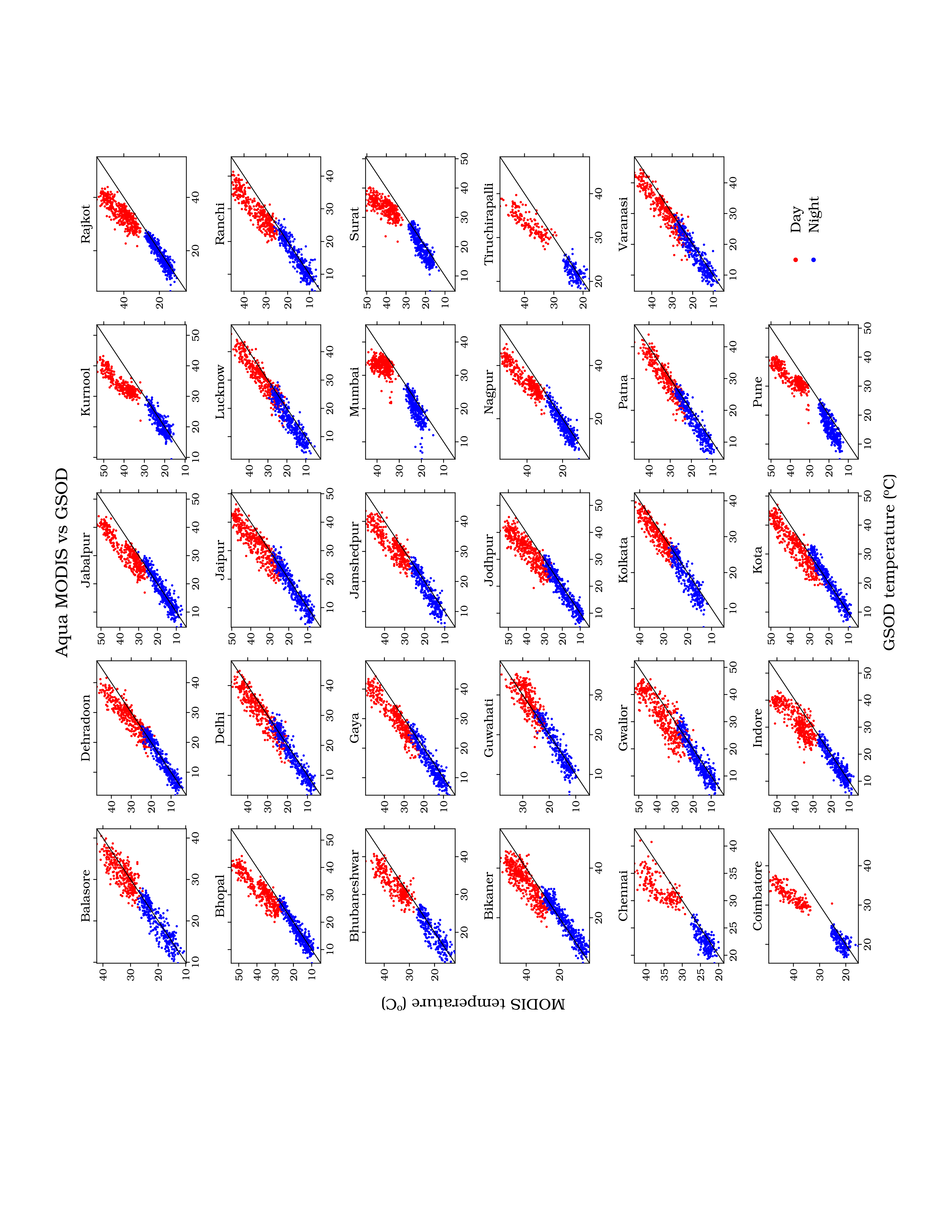


**Figure S9.** Relation between GSOD temperatures with respect to LST observed from Aqua satellite for both day and night time represented by scatters plots for 29 available cities. Figure was created using Generic Mapping Tools version 5.4.2 (GMT: http://gmt.soest.hawaii.edu).


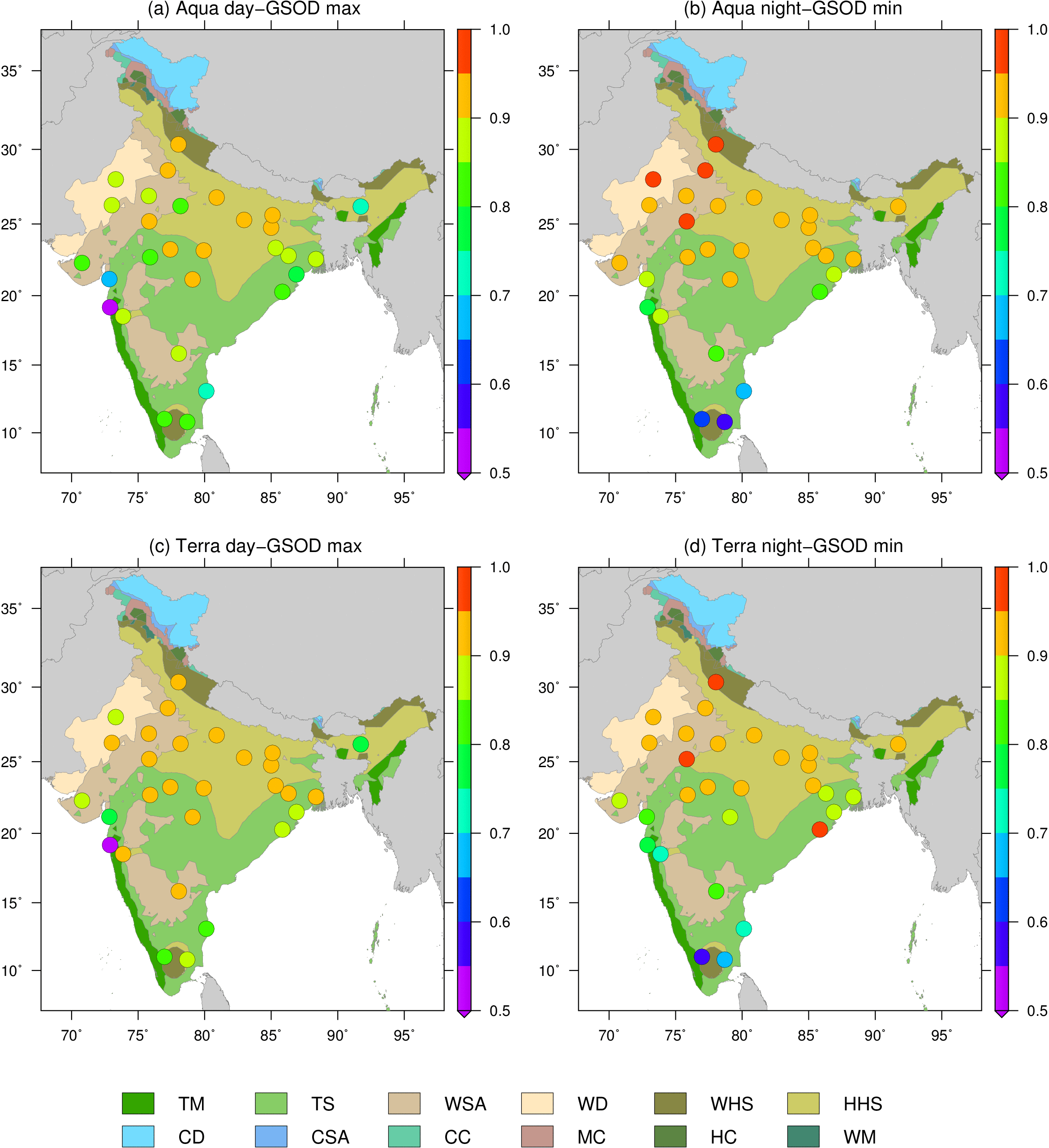


**Figure S10.** Correlation values for GSOD temperature with city core surface temperature for (a) aqua day, (b) aqua night, (c) terra day and (d) terra night with respective configuration GSOD temperatures for 29 available cities. Figure was created using Generic Mapping Tools version 5.4.2 (GMT: http://gmt.soest.hawaii.edu).


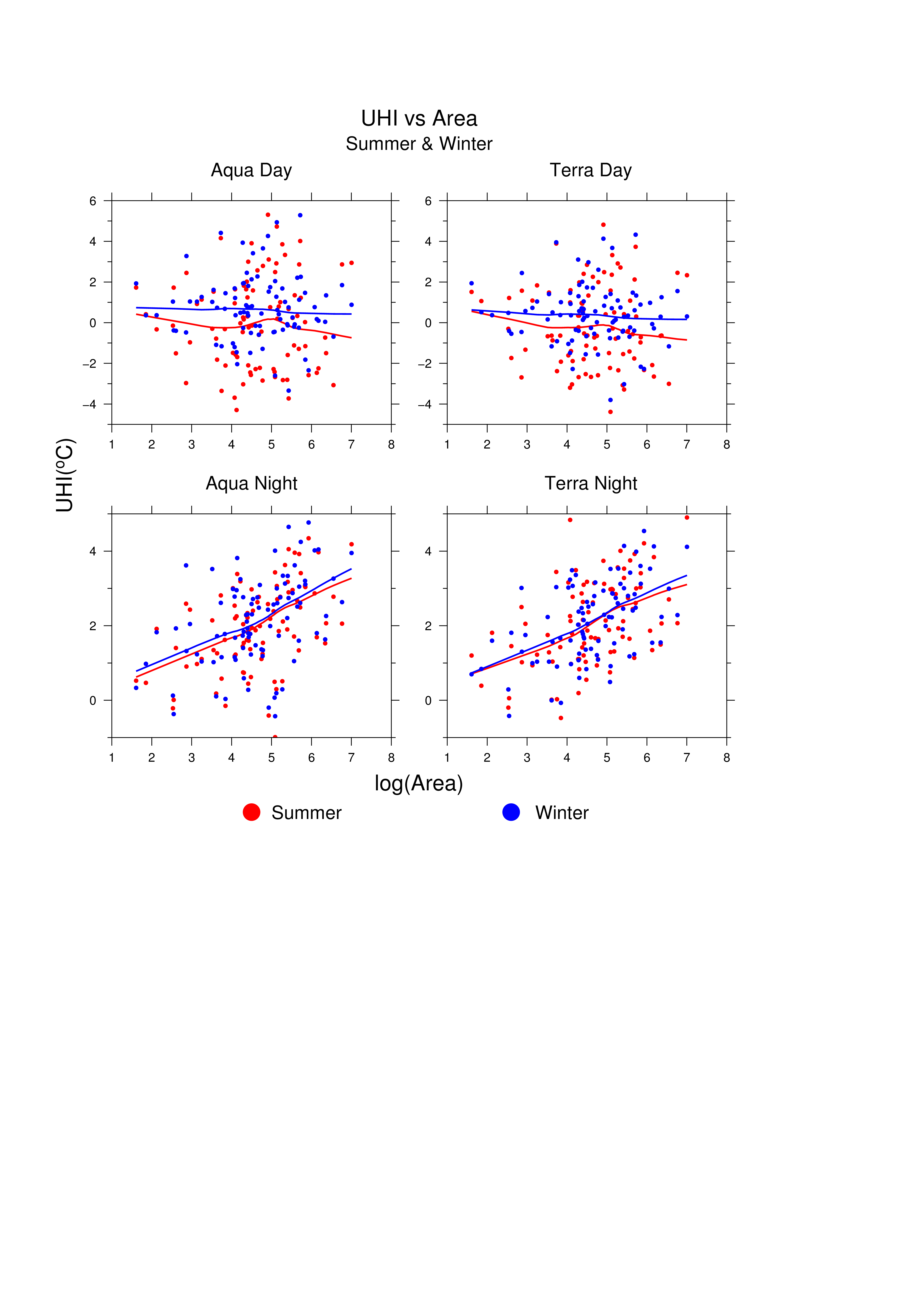


**Figure S11.** Relation between median UHI values in ^o^C of each city with respective log(Area) in km^2^. The scatter values were fitted with loess fit lines. Area of the city is as available from Schneider et al. (2009). Figure was created using Generic Mapping Tools version 5.4.2 (GMT: http://gmt.soest.hawaii.edu).


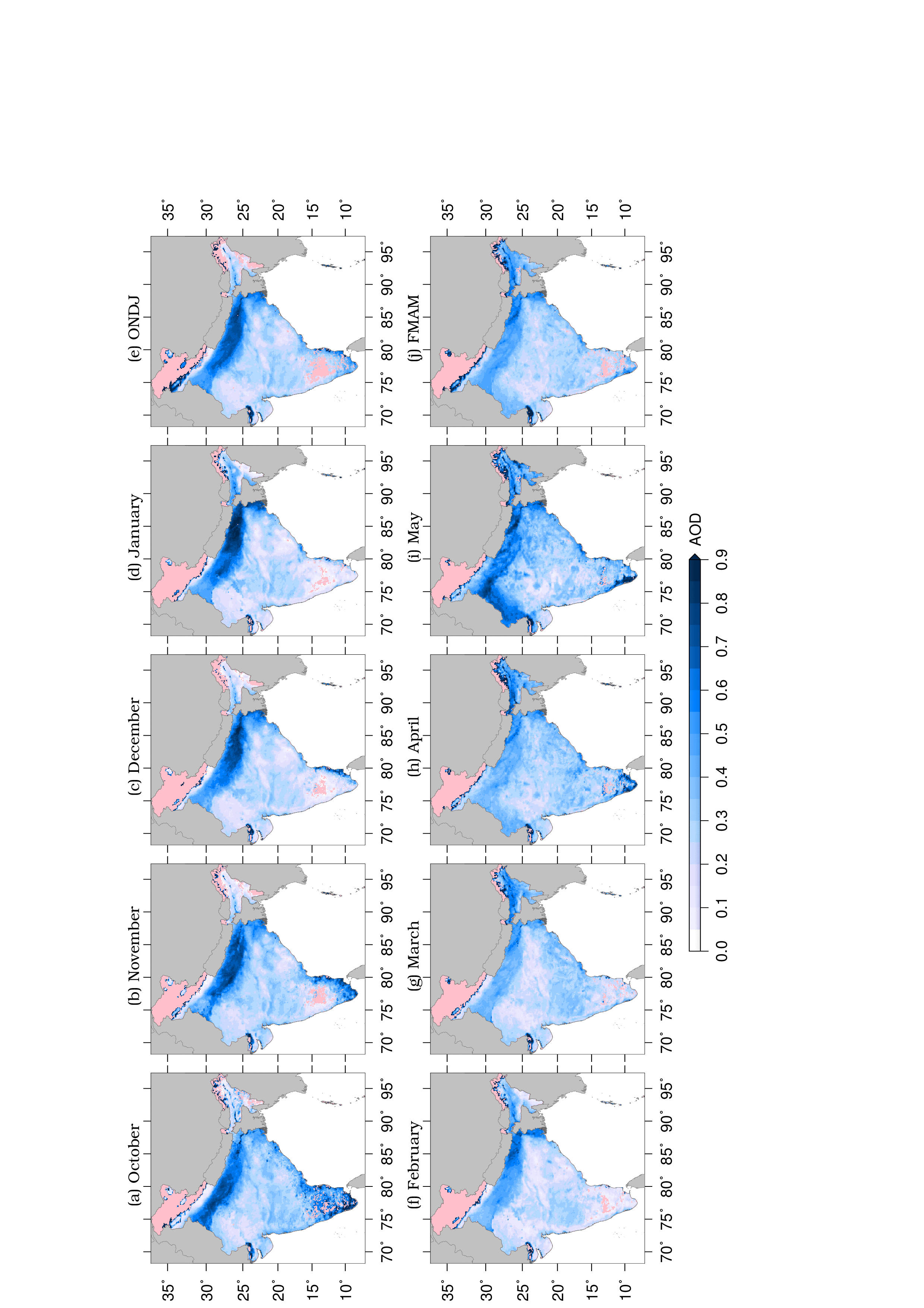


**Figure S12.** Median monthly AOD for the period 2003-2014 as obtained from Aqua MODIS platform. Pink pixels represents considerable missing data. Figure was created using Generic Mapping Tools version 5.4.2 (GMT: http://gmt.soest.hawaii.edu).


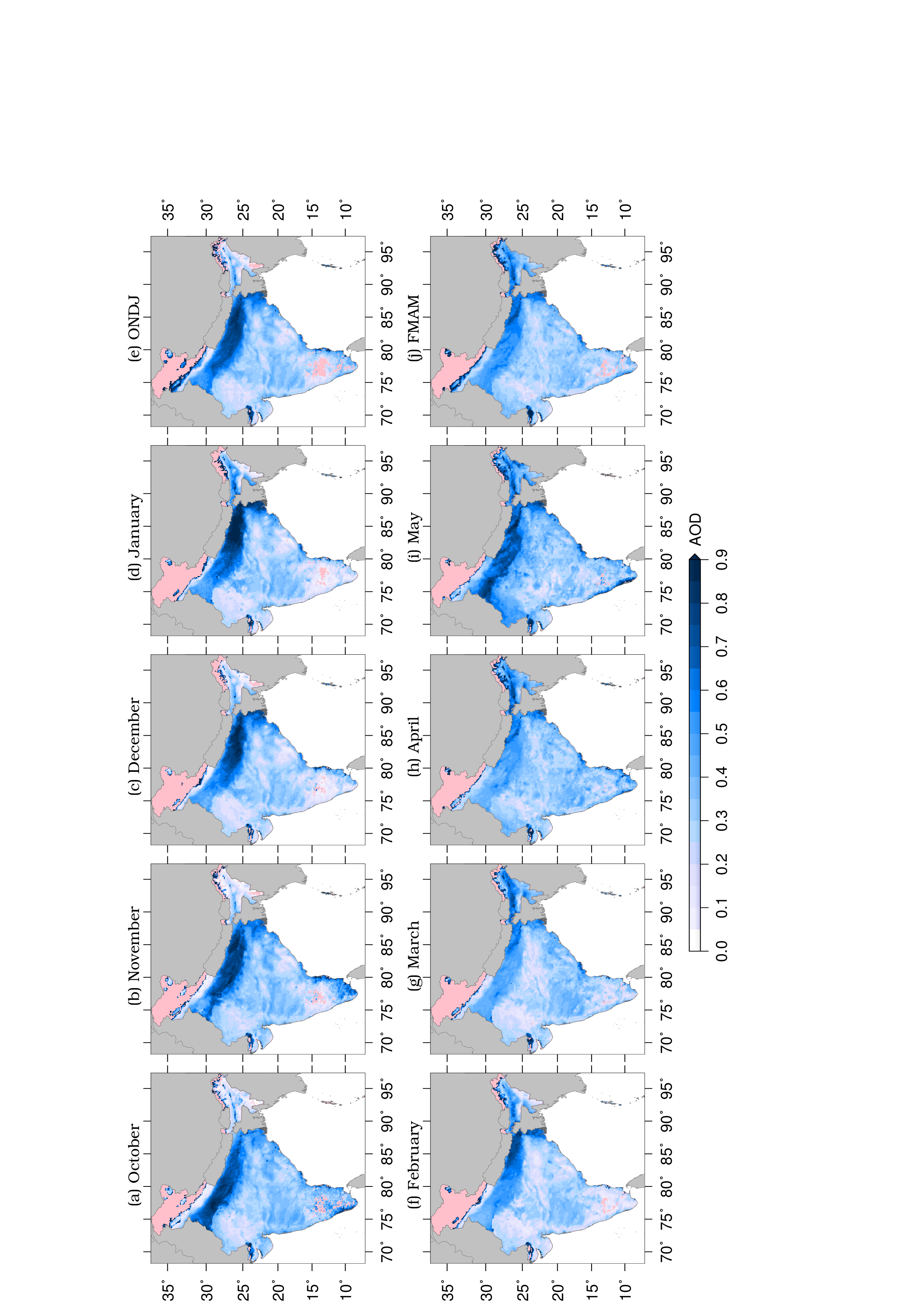


**Figure S13.**  Median monthly AOD for the period 2003-2014 as obtained from Terra MODIS platform. Pink pixels represents considerable missing data. Figure was created using Generic Mapping Tools version 5.4.2 (GMT: http://gmt.soest.hawaii.edu).


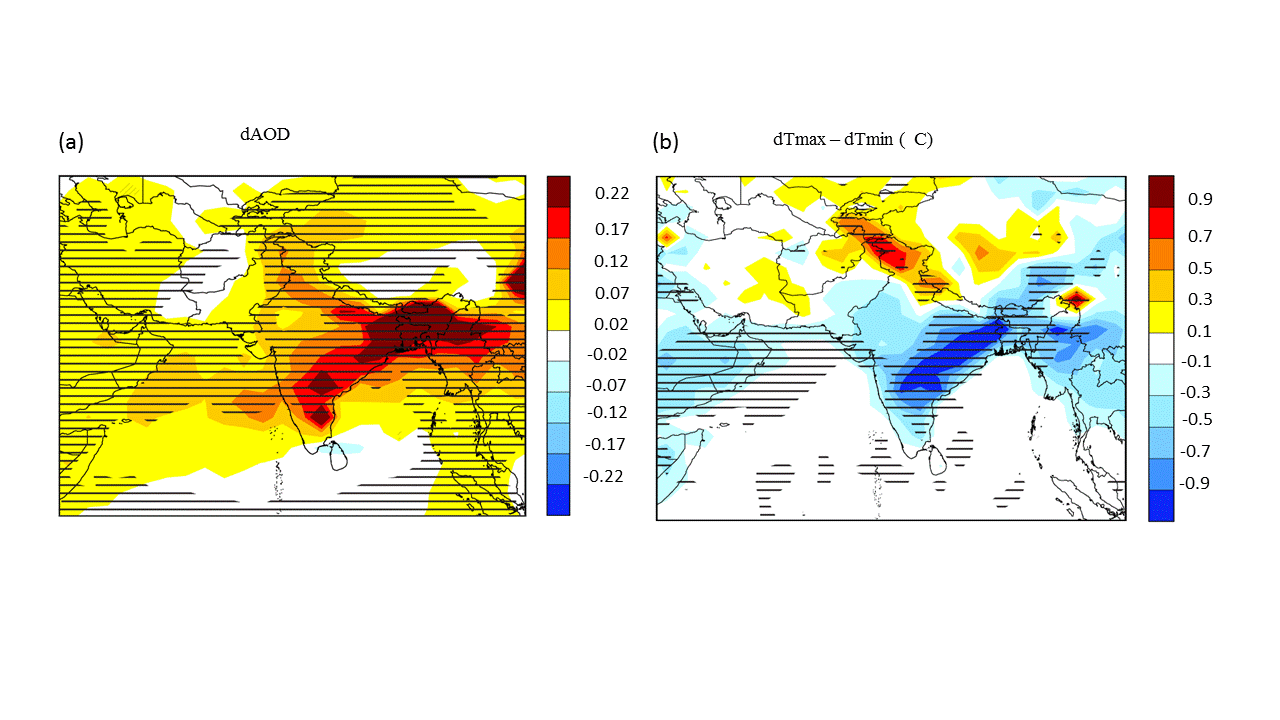


**Figure S14.** Changes in Aerosol Optical Depth (dAOD) and corresponding change (^o^C) in the difference of daily maximum and minimum air temperatures (dTmax-dTmin). Hatching indicates results are statistically significant at the 95% confidence interval based on unforced interannual variability within the model. Figure was created using Generic Mapping Tools version 5.4.2 (GMT: http://gmt.soest.hawaii.edu).

**Supplementary Data References**

1. Schneider, A., Friedl, M. A. & Potere, D. A new map of global urban extent from MODIS satellite data. *Environ. Res. Lett.* **4,** 44003 (2009).

2. Peel, M. C., Finlayson, B. L. & McMahon, T. a. Updated world map of the K ̈oppen-Geiger climate classification. *Meteorol. Zeitschrift* **15,** 259–263 (2006).
